# *In Vivo* Linking of Membrane Lipids and the Anion Transporter Band 3 with Thiourea-modified Amphiphilic Lipid Probes

**DOI:** 10.1038/srep17427

**Published:** 2015-11-30

**Authors:** Akihiro Moriyama, Naohiro Katagiri, Shinichi Nishimura, Nobuaki Takahashi, Hideaki Kakeya

**Affiliations:** 1 Department of System Chemotherapy and Molecular Sciences, Division of Bioinformatics and Chemical Genomics, Graduate School of Pharmaceutical Sciences, Kyoto University, Sakyo-ku, Kyoto 606-8501, Japan

## Abstract

Membrane proteins interact with membrane lipids for their structural stability and proper function. However, lipid–protein interactions are poorly understood at a molecular level especially in the live cell membrane, due to current limitations in methodology. Here, we report that amphiphilic lipid probes can be used to link membrane lipids and membrane proteins *in vivo*. Cholesterol and a phospholipid were both conjugated to a fluorescent tag through a linker containing thiourea. In the erythrocyte, the cholesterol probe fluorescently tagged the anion transporter band 3 via thiourea. Tagging by the cholesterol probe, but not by the phospholipid probe, was competitive with an anion transporter inhibitor, implying the presence of a specific binding pocket for cholesterol in this ~100 kDa protein. This method could prove an effective strategy for analyzing lipid–protein interactions *in vivo* in the live cell membrane.

How membrane lipids and membrane proteins interact with each other is a fundamental question in biology, as lipid–protein (LP) interactions are expected to play important roles in the cell membrane^1^,^2^,^3^. Biochemical analyses have unveiled specific functions of membrane lipids; for example, conferring thermal stability to proteins and modulating the affinity between proteins and ligands. However, membrane lipids can regulate protein properties either directly by binding to a specific pocket, or indirectly by changing the physical properties of the membrane surrounding the protein, such as the curvature, lateral pressure, and bilayer thickness. So far, several dozen membrane proteins have been found to interact with membrane lipids through the analysis of X-ray crystal structures^4^. Recently, ion-mobility mass spectrometry was used to screen for lipids that specifically bind to membrane proteins and thereby increase structural stability^5^. However, we have to carefully interpret data regarding the physiological role of the LP interactions detected *in vitro*, whereas confirming these membrane lipid interactions with proteins in the live cell membrane remains technically challenging.

For linking small molecule ligands and their cellular receptors, chemical strategies utilizing various reactive groups have been employed^6^,^7^,^8^,^9^,^10^. Active site–directed irreversible inhibitors have been used to label target proteins^7^, and are sometimes used to tag shared mechanistic and structural features of large enzyme classes; for example, fluorophosphonates can tag serine hydrolase family proteins^8^,^9^. On the other hand, the decoration of high-affinity ligands with reactive functions enables labeling of the receptor molecule in the proximity of the active site. The quantity, activity, and subcellular localization of the corresponding protein can therefore be detected using these approaches. Theoretically, such irreversible–modification approaches may be applicable to membrane lipids for detecting LP interactions. So far, a couple of photoreactive lipid probes have been utilized for global analysis of *in vivo* LP interactions^11^,^12^,^13^. However, the potential of lipid probes possessing reactive groups other than photoreactive functions has not been explored. Here we demonstrate that the chemical tagging approach using thiourea as a reactive function can indeed be used to link lipids and the anion transporter band 3 in the erythrocyte membrane. This approach enabled us to identify a putative binding pocket of cholesterol in a large ~100 kDa membrane protein.

## Results

### Chemical tagging of band 3 by a cholesterol probe

**Figure 1:**
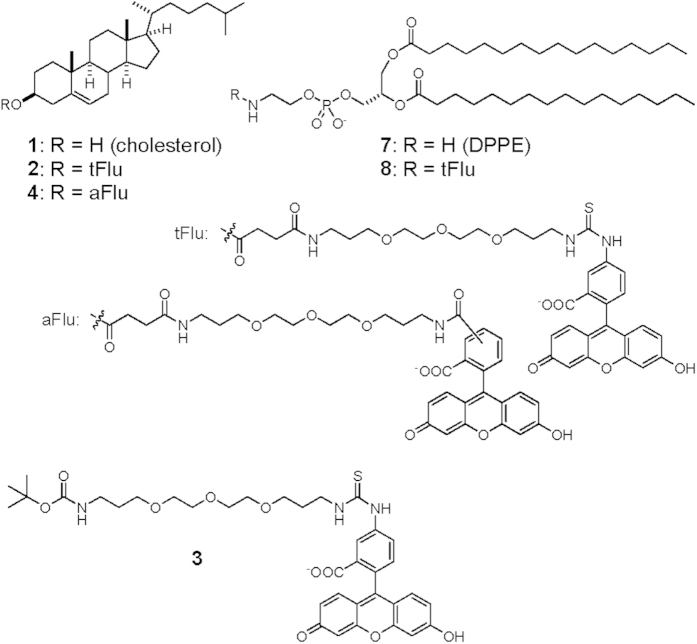
Chemical structures of lipids and lipid probes.

**Figure 2:**
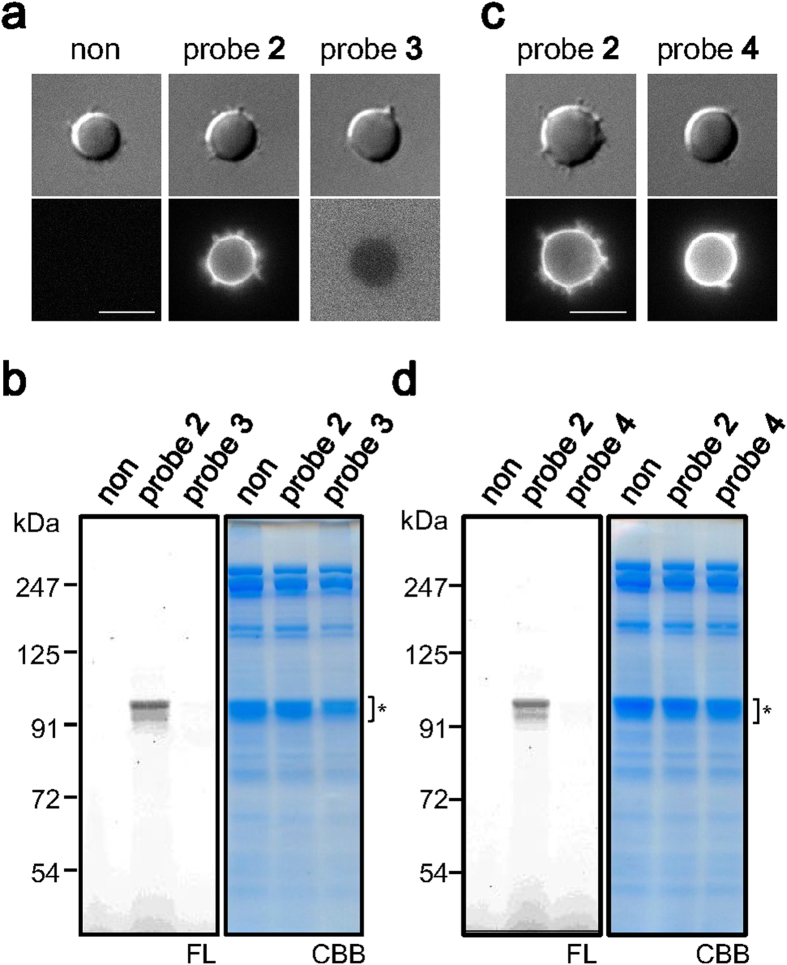
Tagging of the anion transporter band 3 by the cholesterol probe 2. (**a,b**) Binding and tagging with the cholesterol probe **2**. Erythrocytes were treated with probe **2** or **3** (400 nM) at 37 °C, and observed under fluorescence microscopy (**a**). Bright field (DIC) and fluorescent images are shown. Scale bar, 5 μm. After 8 h incubation, cells were lysed and analyzed by SDS-PAGE (**b**). The fluorescence and CBB images of the gel are shown. Asterisk indicates band 3. (**c,d**) Erythrocytes were treated with the cholesterol probe **2** or **4**, and analyzed as in (**a,b**).

Cholesterol (**1**), the key lipid species in regulating the membrane order^14^, likely modulates protein stability and function in general^15^. There have been reports of cholesteryl ethers with polyethylene glycol (PEG) forming liposomes^16^ or micelles^17^,^18^ in aqueous solutions. Such amphiphilic molecules can be used as cholesterol probes with no membrane permeability, e.g. fluorescein-labeled PEG-cholesterol molecules with a PEG repeat of 50 or 200 were shown to distribute to cholesterol-rich domains in the outer leaflet of the plasma membrane^18^,^19^. We designed and synthesized a cholesterol probe (**2**) and surveyed the interactions of cholesterol in the sheep erythrocyte (Fig. 1). The cholesterol probe **2** has a short linker for chemically tagging amino acids near the cholesterol binding site. We chose thiourea as the reactive moiety because of its mild reactivity. As expected, the fluorescence of probe **2** was observed mainly on the cell surface, when erythrocytes were treated with the probe (Fig. 2a). The treated erythrocytes were lysed and subjected to SDS-PAGE, followed by fluorescence imaging. We detected tagging of a ~100 kDa protein with high specificity (Fig. 2b). In contrast, the control probe **3**, in which cholesterol was replaced with a Boc group, neither bound to the cells nor tagged the protein (Fig. 2a,b). Judging from the molecular weight and the SDS-PAGE pattern, the tagged protein was identified as the anion transporter band 3, which was subsequently confirmed by LC-MS/MS analysis (Figure S1).

**Figure 3:**
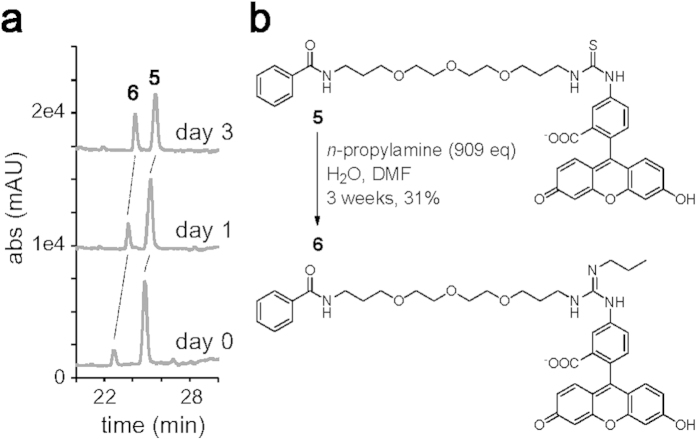
Reactivity of thiourea with amine. (**a**) Time course analysis of the reaction converting compound **5** to **6**. Compound **5** was incubated with *n*-propylamine (3473 eq) and analyzed by LC-MS. UV chromatograms at 488 nm are shown. (**b**) Chemical structures of model compounds **5** and **6**. The thiourea compound **5** was converted to the guanidine compound **6** in the presence of excess *n*-propylamine.

Band 3 is the most abundant protein in the erythrocyte membrane (30% in human erythrocytes)^20^. No other protein bands tagged by the cholesterol probe **2** were detectable. The tagging level saturated after 24 h incubation in the presence of 400 nM of probe **2**, and a linear correlation between the tagging level and the probe concentration was observed below 800 nM using an 8 h incubation period (Figure S2). The tagging level was tentatively estimated to be 0.073% when incubated with 400 nM of probe **2** for 8 h (Figure S2). This low-level tagging was likely due to mild-reactivity of thiourea (see Fig. 3). The abundance of endogenous cholesterol in the erythrocyte membrane can also contribute to the low efficiency; the quantity of cholesterol in human erythrocytes is 36 nmol/10 e8 cells^21^, whereas 0.4 nmol of probe **2** was incubated with 10 e8 cells in our experiments. Significant competition would therefore have been present between the abundant endogenous cholesterol and the cholesterol probe. In addition, there is a possibility that probe **2** can access band 3 in a specific conformation only, or in a specific complex; band 3 is known to exist in at least three populations, including the ankyrin-associated complex, the junctional complex, and as free dimers^22^,^23^, and within each class are subpopulations with further structural heterogeneity^24^.

### Reactivity of thiourea with amine

To our knowledge, thiourea has never previously been utilized for cross-linking biological molecules. The involvement of thiourea was investigated by synthesizing another cholesterol probe **4** in which an amide group was used to conjugate fluorescein to the linker. Probe **4** bound to the erythrocytes to a level comparable to that of probe **2** (Fig. 2c). However, no tagging of band 3 was detected, thus confirming the reactivity of thiourea in the cell membrane (Fig. 2d). We next synthesized a model compound and analyzed the reactivity of thiourea under aqueous conditions. When we mixed the model molecule **5** with an excess amount of *n*-propylamine, the amount of **5** gradually decreased to give rise to a new peak (**6**) observed by LC-MS analysis (Fig. 3). We purified the newly-emerged compound **6** and analyzed its structure by MS and NMR spectra. The ion peak in ESI MS revealed the molecular formula of C_41_H_46_N_4_O_9_, which lacked the S atom and had additional C_3_H_7_N compared with **5** (*m/z* 739.3334 [M + H]^+^, Δmmu = 0.4, calcd for C_41_H_47_N_4_O_9_). In addition, the ^13^C NMR spectrum of compound **6** did not show a quaternary carbon signal (*δ*_C_ 182.5 ppm) corresponding to the thiourea carbon in compound **5**. Instead, a new carbon signal (*δ*_C_ 156.4 ppm) and a set of signals corresponding to the propyl group were detected. These data indicated that thiourea and *n*-propylamine reacted to yield guanidine (Fig. 3), which was supported by the fully assigned ^1^H NMR data. Because the reactivity of thiourea was low, the specific tagging by probe **2** led us to hypothesize that cholesterol binds with high affinity to band 3.

**Figure 4:**
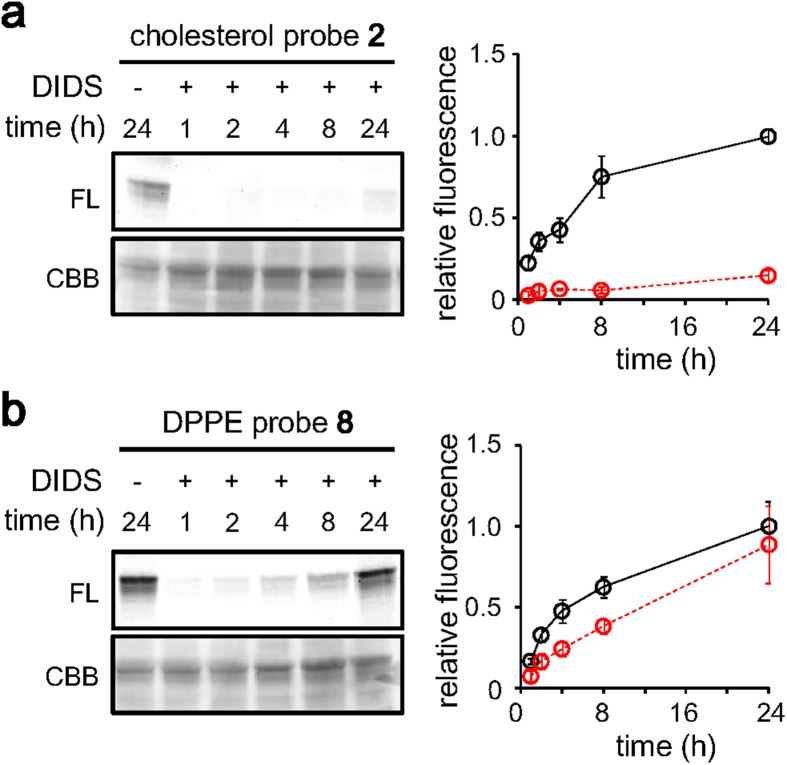
Competitive tagging of band 3 by lipid probes. Erythrocytes were pre-treated with a transporter inhibitor DIDS (100 μM) for 30 min, then time-dependent tagging was examined using the cholesterol probe **2** (**a**) or the DPPE probe **8** (**b**). The concentration of the probes was 400 nM. The fluorescence intensities relative to that obtained after 24 h incubation in the absence of DIDS are shown. The tagging level in the absence (black solid line, Figures S2 and S4) or in the presence (red dashed line) of DIDS is shown. Means ± SD of 3–6 experiments are shown.

**Figure 5:**
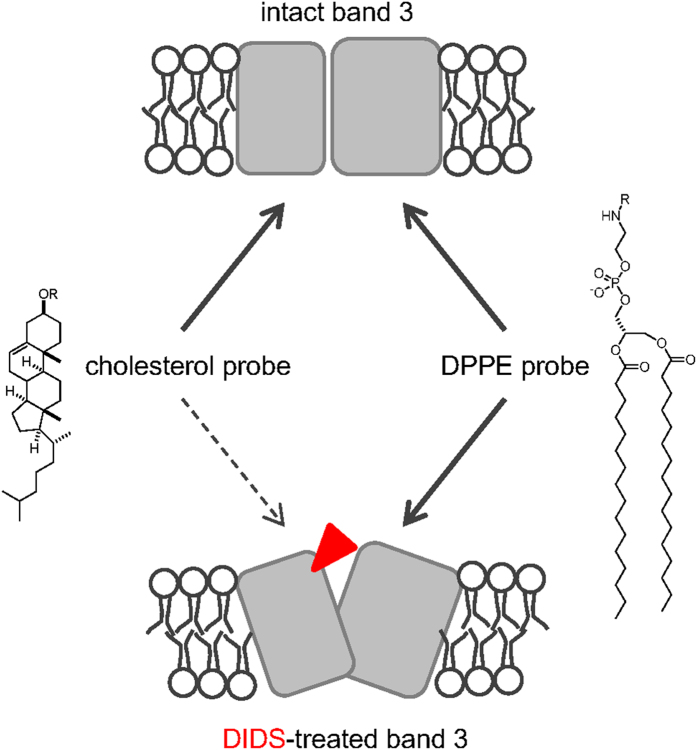
Schemematics for the lipid-band 3 interactions. Both cholesterol and DPPE probes can tag band 3. After DIDS treatment (red triangle), only tagging by cholesterol probe was abolished. Covalent modification of Lys557 by DIDS might directly inhibit tagging of the lysine by the cholesterol probe. Otherwise, overall conformational change by DIDS might inhibit interaction between cholesterol probe and band 3.

Biophysical analyses had predicted the strong binding of cholesterol to band 3, an interaction that may regulate the structure and transporter activity of this protein^25^,^26^,^27^. So far however, nothing has been reported regarding the location of the binding pocket. To investigate the tagging site of the cholesterol probe **2**, a competitive tagging assay using 4,4′-diisothiocyanostilbene-2,2′-disulfonic acid (DIDS), an anion transporter inhibitor, was examined. When erythrocytes were treated with DIDS, we found that tagging by cholesterol probe **2** was significantly suppressed (85% inhibition after 24 h incubation) (Fig. 4a). DIDS is known to form covalent bonds with two lysine residues, e.g. Lys 539 and Lys851 in human band 3, although Lys851 is only reactive at high pH (>8)^28^. In sheep erythrocytes, Lys539 is conserved (Lys557 in sheep), whereas Lys851 is replaced by Arg869, suggesting that Lys557 reacted with thiourea in the cholesterol probe **2** (Fig. 5, **S1**). Alternatively, tagging by cholesterol probe **2** may be inhibited allosterically by DIDS. In fact, band 3 is expected to be fixed in an outward conformation by stilbene compounds such as DIDS^29^, which likely inhibit anion transport activity in an allosteric manner^30^. It is noted that cysteine, another nucleophilic amino acid residue, was not necessary for tagging band 3 since pretreatment of erythrocytes with excess amount of *N*-ethylmaleimide did not inhibit it (Figure S3).

### Ligand-dependent tagging of band 3

We finally expanded our approach to another membrane lipid, 1,2-dipalmitoyl-*sn*-glycero-3-phosphoethanolamine (DPPE) (**7**). We designed probe **8** with the same structural features as that of the cholesterol probe **2**. The DPPE probe **8** bound to erythrocytes to a similar level as cholesterol probes **2** and **4** (Figure S4). Interestingly, probe **8** also specifically tagged band 3 (Fig. 4b**, S4**). The kinetics of the tagging was similar to that of the cholesterol probe **2** (Figure S4), while the tagging efficiency was slightly higher than that of probe **2** (0.21% vs 0.073%) (Figure S2). However, in this case, the competitive effect of DIDS was minor (11.3% inhibition at 24 h; Fig. 4b). This suggested that the binding site of the DPPE probe **8** is not overlapped with the DIDS binding site, or not affected by the conformational change of band 3 by DIDS (Fig. 5). Otherwise, there might be multiple hydrophobic pockets for probe **8**, and the overall tagging level was not decreased upon binding of DIDS. Although we can only speculate the binding mode of the DPPE probe **8**, it is clear that band 3 has multiple lipid binding sites that were occupied by lipid probes **2** and **8**. The atomic basis of these interactions would be more precisely analyzed by X-ray crystal structure analysis.

## Discussion

The approach presented here to link lipids and proteins in the cell membrane has several unique features. First, the lipid probes we used are amphiphilic. Amphiphilicity can suppress membrane permeability, which is a key point when considering the specific tagging of membrane molecules. In fact, when the lipid probes were treated with the erythrocyte lysate, proteins other than band 3 were also tagged (Figure S5). This indicates that the reactivity of thiourea was regulated by the amphiphilicity of the probe, which would enable the introduction of other reactive groups to optimize the tagging rate and the modes of tagging. Next, we applied a novel chemical tagging strategy to the endogenous membrane molecules. Pioneering studies have shown that membrane proteins can be efficiently tagged by chemical probes, which are designed based on soluble inhibitors^31^,^32^. In contrast, our results demonstrated that we could analyze the interactions between endogenous lipids and membrane proteins, in spite of the highly competitive environment *in vivo*. The interactions between cholesterol and membrane proteins are expected to be stabilized by three interactions: hydrophobic interactions, hydrogen bonding interactions, and CH-π interactions^33^,^34^. Although the cholesterol probe **2** cannot form hydrogen bonding due to the absence of the free hydroxyl group at C-3, the hydrophobic and CH-π interactions by cholest-5-en structure in probe **2** could mimic the interaction between cholesterol and proteins. Finally, the thiourea-containing lipid probes revealed a tentative binding site for cholesterol in band 3 (Fig. 5). Although we can speculate that there are multiple interactions between cholesterol and band 3, our cholesterol probes tagged a specific binding site. This might be due to the limited reactivity of thiourea; tagging by thiourea requires the presence of specific amino acids, i.e., lysine, located in the close proximity to the lipid probe. In contrast, cholesterol probes possessing photoreactive diazirine allowed researchers to identify several hundred cholesterol-binding proteins using proteomic analyses^12^. The combination of narrowly-reactive probes for analyzing specific interactions with highly-reactive probes for comprehensive analysis would have significant advantages for LP interactome analyses^35^.

In summary, we have demonstrated that the chemical tagging strategy is feasible for analyzing LP interactions in the cell membrane. Amphiphilic lipid probes can be used to analyze a specific LP interaction, and this may prove applicable to comprehensive proteomic analyses by tuning the probe reactivities. Expanding this approach to exogenous molecules targeting membrane lipids, such as antibiotics, is also an attractive challenge.

## Methods

### General considerations for organic synthesis

All reagents and solvent were used as received from commercial suppliers and were used without further purification. IR spectra were measured using an FTIR spectrometer equipped with a ZnSe ATR plate. Optical rotations were determined using the sodium D line (589 nm). NMR spectra were measured on a 500 MHz instrument. ^1^H and ^13^C chemical shifts (δ) are shown in parts per million (ppm), and coupling constants (*J*) are in hertz (Hz). The following abbreviations are used to describe multiplicities: s, singlet; d, doublet; t, triplet; dd, doublet of doublets; dt, doublet of triplets; tt, triplet of triplets; m, multiplet; br, broad; ovl, overlapped. Mass spectral data were collected with ESI IT-TOF MS. Flash column chromatography was performed over Silica Flash F60 (SiliCycle) using an elution system as described for each experiment. Synthesis procedures and physico-chemical properties are described in the Supplementary Information file.

### General materials and methods for Biochemical/Biological Experiments

Sheep whole blood was purchased from Kohjin Bio Co., Ltd. and used within two weeks after blood collection. Fluorescent images of SDS-PAGE gels were acquired with Typhoon 9400 (GE Healthcare). Fluorescent images of cells were acquired using an Olympus IX81 microscope equipped with UPLSAPO 100× objective (Olympus, Tokyo, Japan).

### Tagging of anion transporter band 3 with probes

Sheep whole blood (0.5 mL) was centrifuged (300 g × 5 min, unless stated) and supernatant was discarded. The precipitated red blood cells (RBC) were washed with 0.7 mL of ice-cold phosphate buffer (10 mM phosphate, 150 mM NaCl, pH 7.4) for 5 times, and re-suspended in 0.5 mL of phosphate buffer. RBC suspensions (1 mL, 10^8^ cells/mL) were mixed with lipid probes (0.4 μM, 0.4% DMF), and incubated at 37 °C for 8 h with continuous mixing. Microscope analysis was performed within 30–60 min after addition of probes. After incubation, RBC suspensions were centrifuged, washed with 1 mL of ice-cold phosphate buffer twice, and mixed with 80 μL of SDS-PAGE sample buffer. After boiling at 100 °C for 5 min, 20 μL of the sample was subjected to SDS-PAGE (8%) and the fluorescent images were acquired (Ex: 488 nm; Em 532 nm.). All proteins were stained by CBB and the signal intensity was calculated using MetaMorph software (Molecular Devices).

### Competition assay with DIDS

Washed RBC solutions (×10^8^ cells/mL) were prepared as described. RBCs were treated with DIDS (final concentration: 100 μM in 0.4% DMF) on ice for 30 min in the dark. After incubation, RBC solution was centrifuged, and washed with phosphate buffer (1 mL) twice followed by washing with a BSA solution (0.5 w/v% BSA in PBS). After extensive PBS wash of RBCs (1 mL each, three times), RBCs were suspended in 1 mL of phosphate buffer and treated with lipid probes as described above.

### Reactivity test of the thiourea-containing model compound 5

Compound **5** (2 mg/mL, 2.8 mM) was mixed with *n*-propylamine (400 μL, 3473 eq), 4,4′-bis(2-methyl-2-propanyl)biphenyl (0.4 mg/mL), DMF (50 μL), and water (50 μL), which was stirred at 37 °C. After incubation, the reaction mixture (10 μL) was mixed with 200 μL of water, lyophilized, and dissolved in 500 μL of DMF. A portion (1 μL) of the prepared sample was subjected to LC-MS analysis. The analytes were separated by an ODS column (Senshu-PAK PEGASIL ODS SP100, *ϕ*3 × 100 mm, 30 °C) using a gradient elution system (50–100% aq MeOH, 200 μL/min).

## Additional Information

**How to cite this article**: Moriyama, A. *et al.* *In Vivo* Linking of Membrane Lipids and the Anion Transporter Band 3 with Thiourea-modified Amphiphilic Lipid Probes. *Sci. Rep.* **5**, 17427; doi: 10.1038/srep17427 (2015).

## Supplementary Material

Supplementary Information
