# Supplementary material for: In Vivo Linking of Membrane Lipids and the Anion Transporter Band 3 with Thiourea-modified Amphiphilic Lipid Probes
Source: Sci Rep. 2015 Nov 30;5:17427. doi: 10.1038/srep17427 (PMC4663539; doi:10.1038/srep17427)

Supplementary Information

In vivo Linking of Membrane Lipids and the Anion Transporter Band 3 with Thiourea-modified Amphiphilic Lipid Probes.

Akihiro Moriyama, Naohiro Katagiri, Shinichi Nishimura,* Nobuaki Takahashi and Hideaki Kakeya*

Department of System Chemotherapy and Molecular Sciences, Division of Bioinformatics and Chemical Genomics, Graduate School of Pharmaceutical Sciences, Kyoto University, Sakyo-ku, Kyoto 606-8501, Japan

*nshin@pharm.kyoto-u.ac.jp (SN); scseigyo-hisyo@pharm.kyoto-u.ac.jp (HK)

Organic synthesis: page S2-S9

Supplementary figures: page S10-S14

1H NMR spectra: page 15-25

Organic synthesis

Synthesis of the cholesterol probe 2.

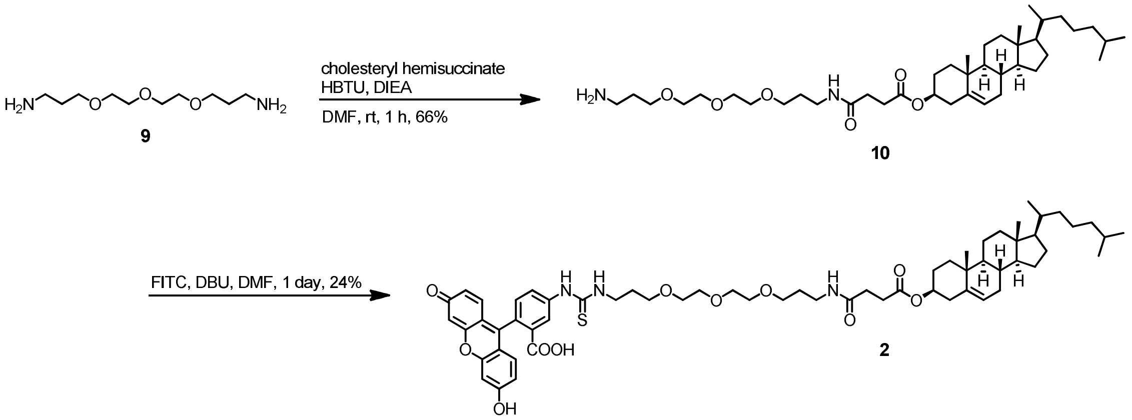

(3S,8S,9S,10R,13R,14S,17R)-10,13-dimethyl-17-((R)-6-methylheptan-2-yl)-2,3,4,7,8,9,10,11,12,13,14,15,16,17-tetradecahydro-1H-cyclopenta[a]phenanthren-3-yl 1-amino-15-oxo-4,7,10-trioxa-14-azaoctadecan-18-oate (**10**)

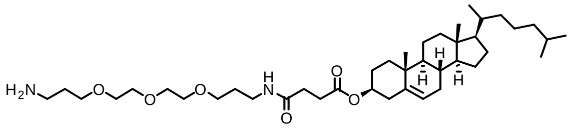

To a stirred solution of cholesteryl hemisuccinate (52.5 mg, 0.107 mmol) in DMF (1.5 mL) were added HBTU (57.0 mg, 0.155 mmol) and DIEA (26.9 μL, 0.155 mmol). After being stirred at room temperature for 30 min, one third of the mixture was added dropwise to a stirred solution of **9** (225.8 μL, 1.03mmol) in DMF (1.5 mL). After 45 min, the remaining portion of the activatead cholesteryl hemisuccinate solution was added to the reaction mixture. After being stirred for 1 h at room temperature, the reaction was quenched with water. Aqueous solution was extracted with $CHCl_3$ which was dried over Na_2SO_4 and concentrated *in vacuo*. The residue was chromatographed on a SiO_2 column with a stepwise elution of EtOAc, and $CHCl_3$/MeOH (20/1, and 1/1). Fractions eluted with $CHCl_3$/MeOH (1/1) were combined and concentrated *in vacuo* to give **10** (49.2 mg, 66%): $[\alpha]_D^{20}$ = -22.3 (c 2.3, $CHCl_3$); IR (neat) 3300, 2934, 2867, 1732, 1652, 1551, 1364, 1254, 1173, 1109, 844 cm^{-1}; ^{1}H NMR ($CDCl_3$, 500 MHz) δ 5.35 (br d, J = 4.2 Hz, 1H), 4.57 (m, 1H), 3.75 (t, J = 5.1 Hz, 2H), 3.67-3.58 (ovl, 8H), 3.55 (t, J = 5.8 Hz, 2H), 3.33 (m, 2H), 3.25 (m, 2H), 2.64 (t, J = 6.0 Hz, 2H), 2.55 (t, J = 6.4 Hz, 2H), 2.30 (m, 2H), 2.06-1.92 (ovl, 4H), 1.88-1.77 (ovl, 4H), 1.64-1.41 (ovl, 7H), 1.41-1.20 (ovl, 6H), 1.19-0.93 (ovl, 10H), 1.01 (s, 3H), 0.91 (d, J = 6.4 Hz, 3H), 0.86 (d, J = 6.6 Hz, 3H), 0.86 (d, J = 6.6 Hz, 3H), 0.67 (s, 3H); HRMS (ESI) *m/z* 689.5472 $[M + H]^+$ calcd for $C_{41}H_{73}N_2O_6$, 689.5463.

5-(3-(18-(((3S,8S,9S,10R,13R,14S,17R)-10,13-dimethyl-17-((R)-6-methylheptan-2-yl)-2,3,4,7,8,9,10,11,12,13,14,15,16,17-tetradecahydro-1H-cyclopenta[a]phenanthren-3-yl)oxy)-15,18-dioxo-4,7,10-trioxa-14-azaoctadecyl)thioureido)-2-(6-hydroxy-3-oxo-3H-xanthen-9-yl)benzoic acid (**2**)

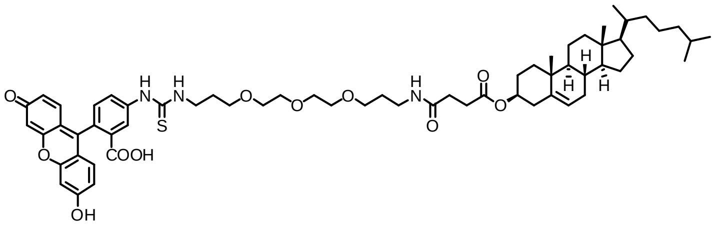

Fluorescein isothiocyanate (FITC) (15.4 mg, 0.0395 mmol), **10** (22.4 mg, 0.0325 mmol), DMF (0.5 mL), and DBU (5.5 μL, 0.0368 mmol) were mixed and stirred in a brown microtube at room temperature for 1 day. The reaction mixture was directly loaded on an ODS open column, and chromatographed with a stepwise elution of H_2O/MeOH (from 100/0 to 0/100). Fractions eluted with H_2O/MeOH (10/90 to 0/100) were combined and concentrated *in vacuo*. The residue was subjected to ODS HPLC on COSMOSIL 5C_8-MS (ϕ 20 × 250 mm) with H_2O/MeCN (5/95) to afford **2** (8.7 mg, 24%): $[\alpha]_D^{20}$ = -10.8 (c 0.73, CH_3OH); UV (MeOH) λ_{max} (log ε) 225 (5.11), 276 (4.75), 453 (4.40), 479 (4.40) nm; IR (neat) 3285, 2932, 2868, 1733, 1609, 1506, 1456, 1329, 1254, 1175, 1105, 849 cm^{-1}; ^1H NMR (CD_3OD, 500 MHz) δ 8.10 (d, J = 1.7 Hz, 1H), 7.77 (d, J = 8.1 Hz, 1H), 7.15 (d, J = 8.3 Hz, 1H), 6.72 (d, J = 8.7 Hz, 2H), 6.66 (d, J = 2.2 Hz, 2H), 6.54 (dd, J = 8.6, 2.3 Hz, 2H), 5.33 (br d, J = 4.4 Hz, 1H), 4.49 (m, 1H), 3.72 (br, 2H), 3.64-3.58 (ovl, 8H), 3.55 (m, 2H), 3.48 (t, J = 6.3 Hz, 2H), 3.23 (t, J = 6.6 Hz, 2H), 2.56 (t, J = 6.7 Hz, 2H), 2.44 (t, J = 6.9 Hz, 2H), 2.27 (br d, J = 7.5 Hz, 2H), 2.01 (m, 1H), 1.95 (m, 1H), 1.92 (tt, J = 6.1, 6.1 Hz, 2H), 1.88-1.77 (ovl, 3H), 1.72 (tt, J = 6.5, 6.5 Hz, 2H), 1.62-1.22 (ovl, 13H), 1.22-0.96 (ovl, 9H), 1.00 (s, 3H), 0.92 (d, J = 6.5 Hz, 3H), 0.87 (d, J = 6.6 Hz, 3H), 0.87 (d, J = 6.6 Hz, 3H), 0.69 (s, 3H); HRMS (ESI) *m/z* 1078.5811 $[M + H]^+$ calcd for $C_{62}H_{84}N_3O_{11}S$, 1078.5821.

Synthesis of the Boc probe 3.

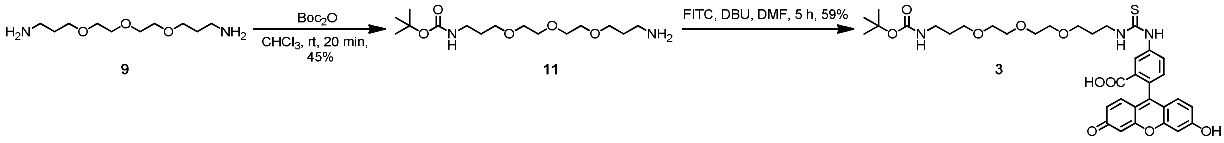

tert-butyl(3-(2-(2-(3-aminopropoxy)ethoxy)ethoxy)propyl)carbamate (**11**)

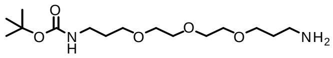

To a stirred solution of **9** (1.0 mL, 4.56 mmol) in $CHCl_3$ (100 mL) was added Boc_2O (727 μL, 3.60 mmol) dripwise. After being stirred at room temperature for 20 min, the reaction was quenched with saturated aqueous $NaHCO_3$. The mixture was extracted with $CHCl_3$, dried over anhydrous Na_2SO_4, and concentrated *in vacuo*. The mixture was chromatographed on a SiO_2 column with a stepwise elution of EtOAc, and $CHCl_3$/MeOH (1/1). Fractions eluted with $CHCl_3$/MeOH (1/1) were combined and concentrated *in vacuo* to afford **11** as a light yellow oil (523.2 mg, 45%): IR (neat) 3355, 2927, 2866, 1697, 1519, 1390, 1364, 1170, 1105, 861 cm^{-1}; ^1H NMR ($CDCl_3$, 500 MHz) δ 3.62-3.59 (br, 2H), 3.57-3.52 (ovl, 8H), 3.48 (t, J = 6.4 Hz, 2H), 3.10 (t, J = 6.9 Hz, 2H), 2.72 (t, J = 6.9 Hz, 2H), 1.71 (tt, J = 6.4, 6.4 Hz, 2H), 1.69 (tt, J = 6.4, 6.4 Hz,

2H), 1.41 (s, 9H); ^{13}C NMR ($CDCl_3$, 125 MHz) δ 156.1, 78.8, 70.6, 70.3, 70.2, 69.6, 69.5, 39.6, 38.5, 33.2, 30.2, 29.7, 28.5 ; HRMS (ESI) *m/z* 321.2385 $[M + H]^+$ calcd for $C_{15}H_{33}N_2O_5$, 321.2384

5-(3-(2,2-dimethyl-4-oxo-3,9,12,15-tetraoxa-5-azaoctadecan-18-yl)thioureido)-2-(6-hydroxy-3-oxo-3H-xanthen-9-yl)benzoic acid (**3**)

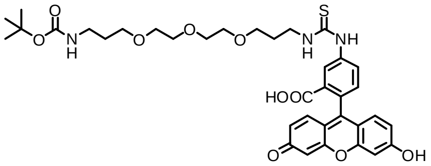

FITC (29.8 mg, 0.0765 mmol), **11** (23.9 mg, 0.0745 mmol), DMF (0.5 mL), and DBU (11.2 μL, 0.0745 mmol) were mixed and stirred in a brown microtube at room temperature for 5 h. The reaction mixture was directly loaded on an ODS open column, and chromatographed with a stepwise elution of H_2O/MeOH (from 100/0 to 0/100). Fractions eluted with H_2O/MeOH (70/30 to 0/100) were combined and concentrated *in vacuo*. A portion of the residue (16.2 mg of 65.34 mg) was subjected to reversed-phase HPLC (PEGASIL ODS SP100, ϕ 20 × 250 mm, H_2O/MeOH (from 70/30 to 0/100)) to afford **3** (7.71 mg, 59%): UV (MeOH) λ_{max} (log ε) 224 (5.28), 275 (4.90), 453 (4.51), 479 (4.51) nm; IR (neat) 3287, 2929, 2871, 1739, 1682, 1608, 1505, 1454, 1173, 1111, 850 cm^{-1}; ^1H NMR (CD_3OD, 500 MHz) δ 8.09 (s, 1H), 7.73 (d, *J* = 8.1 Hz, 1H), 7.14 (d, *J* = 8.1 Hz, 1H), 6.72 (d, *J* = 8.6 Hz, 2H), 6.65 (d, *J* = 1.8 Hz, 2H), 6.54 (dd, *J* = 8.6, 1.8 Hz, 2H), 3.60-3.58 (ovl, 8H), 3.53 (m, 2H), 3.46 (t, *J* = 6.3 Hz, 2H), 3.29 (m, 2H), 3.08 (t, *J* = 6.3 Hz, 2H), 1.90 (tt, *J* = 6.3, 6.3 Hz, 2H), 1.67 (tt, *J* = 6.3, 6.3 Hz, 2H), 1.40 (s, 9H); HRMS (ESI) *m/z* 710.2744 $[M + H]^+$ calcd for $C_{36}H_{44}N_3O_{10}S$, 710.2742.

Synthesis of probe 4.

4-((18-(((3S,9S,10R,13R,14S,17R)-10,13-dimethyl-17-((S)-6-methylheptan-2-yl)-2,3,4,7,8,9,10,11,12,13,14,15,16,17-tetradecahydro-1H-cyclopenta[a]phenanthren-3-yl)oxy)-15,18-dioxo-4,7,10-trioxa-14-azaoctadecyl)carbamoyl)-2-(6-hydroxy-3-oxo-3H-xanthen-9-yl)benzoic acid and 5-((18-(((3S,9S,10R,13R,14S,17R)-10,13-dimethyl-17-((S)-6-methylheptan-2-yl)-2,3,4,7,8,9,10,11,12,13,14,15,16,17-tetradecahydro-1H-cyclopenta[a]phenanthren-3-yl)oxy)-15,18-dioxo-4,7,10-trioxa-14-azaoctadecyl)carbamoyl)-2-(6-hydroxy-3-oxo-3H-xanthen-9-yl)benzoic acid (**4**)

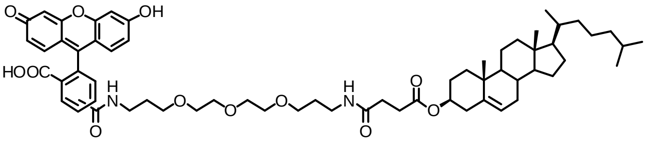

Cholesterol-derived amine **10** (5.0 mg, 7.26 × 10^{-3} mmol), NHS-fluorescein (5.5 mg, 1.15 × 10^{-2} mmol), 0.5 mL of DMF, and DBU (1.08 μL, 7.22 × 10^{-3} mmol) were mixed and stirred in a brown microtube at room temperature for 3 h. The mixture was directly chromatographed on an ODS open column with a stepwise elution of H_2O/MeOH (from 40/60 to 0/100). Fractions eluted with H_2O/MeOH (0/100) were combined and

concentrated *in vacuo*. The residue was subjected to reverse-phase HPLC (COSMOSIL $5C_8$-MS, ϕ 20 × 250 mm, H_2O/MeOH (5/95)) to give **4** (3.0 mg, 39%): UV (MeOH) λ_{max} (log ε) 225 (4.84), 273 (4.16), 454 (4.20), 481 (4.19) nm; IR (neat) 3279, 2933, 2870, 1734, 1643, 1608, 1506, 1465, 1382, 1249, 1171, 1111, 850 cm^{-1}; ^1H NMR (CD_3OD, 500 MHz) δ 8.17 (dd, J = 7.9, 1.5 Hz, 0.53H), 8.13 (dd, J = 8.0, 1.1 Hz, 0.47H), 8.06 (d, J = 8.0 Hz, 0.47H), 7.88 (m, 0.47H), 7.63 (s, 0.53H), 7.29 (d, J = 8.0 Hz, 0.53H), 6.69-6.60 (ovl, 4H), 6.54 (m, 2H), 5.33 (br, 1H), 4.47 (m, 1H), 3.65 (br, 2H), 3.61 (m, 2H), 3.54-3.47 (ovl, 8H), 3.41 (m, 2H), 3.19 (m, 2H), 2.53 (td, J = 7.4, 6.9 Hz, 2H), 2.42 (td, J = 7.4, 6.9 Hz, 2H), 2.27 (br d, J = 6.3 Hz, 2H), 2.02 (m, 1H), 2.00 (m, 1H), 1.92 (tt, J = 6.3, 6.3 Hz, 2H), 1.86-1.77 (olv, 3H), 1.68 (tt, J = 6.3, 6.8 Hz, 2H), 1.59-1.25 (ovl, 13H), 1.17-0.99 (ovl, 9H), 0.99 (s, 1.5H), 0.99 (s, 1.5H), 0.92 (d, J = 6.3 Hz, 3H), 0.87 (d, J = 6.9 Hz, 3H), 0.86 (d, J = 6.9 Hz, 3H), 0.69 (s, 1.5H), 0.68 (s, 1.5H); HRMS (ESI) *m/z* 1047.5944 $[M + H]^+$ calcd for $C_{62}H_{83}N_2O_{12}$, 1047.5941

Synthesis of compound 5.

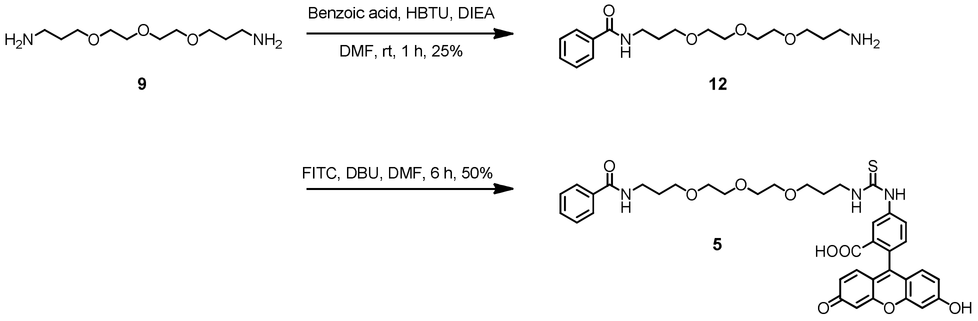

N-(3-(2-(2-(3-aminopropoxy)ethoxy)ethoxy)propyl)benzamide (**12**)

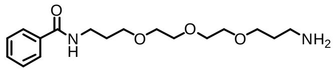

To a stirred solution of benzoic acid (41.0 mg, 0.336 mmol) in DMF (5 mL) were added HBTU (192.96 mg, 0.509 mmol) and DIEA (86.4 μL, 0.496 mmol). After being stirred at room temperature for 30 min, one third of the solution was added to a stirred solution of **9** (719 μL, 3.28 mmol) in DMF (5 mL). The remaining portion of the benzoic acid solution was added to the reaction mixture, which was stirred for 1 h at room temperature. The reaction was quenched with water and concentrated *in vacuo*. The residue was chromatographed on a SiO_2 column with a stepwise elution of $CHCl_3$, and $CHCl_3$/MeOH (20/1, 10/1 and 1/1). Fractions eluted with $CHCl_3$/MeOH (10/1, and 1/1) were combined and concentrated *in vacuo* to give **12** as a yellow oil (27.4 mg, 25%): IR (neat) 3291, 2925, 2871, 1636, 1603, 1540, 1489, 1095, 840 cm^{-1} ; ^1H NMR (CD_3OD, 500 MHz) δ 7.79 (d, J = 6.9 Hz, 2H), 7.51 (t, J = 6.9 Hz, 1H), 7.44 (t, J = 6.9 Hz, 2H), 3.63-3.56 (ovl, 8H), 3.52 (m, 2H), 3.46 (t, J = 6.3 Hz, 2H), 3.29 (m, 2H), 2.71 (t, J = 6.9 Hz, 2H), 1.87 (tt, J = 6.3, 6.3 Hz, 2H), 1.70 (tt, J = 6.3, 6.3 Hz, 2H); ^{13}C NMR (CD_3OD, 125 MHz) δ 168.8, 134.5, 131.3, 128.2, 126.9, 70.2, 70.0, 69.8, 69.1, 68.9, 38.8, 37.4, 31.9, 29.1; HRMS (ESI) *m/z* 325.2108 $[M + H]^+$ calcd for $C_{17}H_{29}N_2O_4$, 325.2122.

2-(6-hydroxy-3-oxo-3H-xanthen-9-yl)-5-(3-(1-oxo-1-phenyl-6,9,12-trioxa-2-azapentadecan-15-yl)thioureido)benzoic acid (**5**)

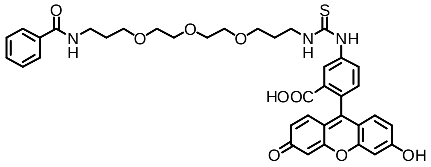

FITC (34.9 mg, 0.0897 mmol), **12** (27.4 mg, 0.0846 mmol), DMF (1 mL), and DBU (12.7 μL, 0.0846 mmol) were mixed and stirred in a brown microtube at room temperature for 6 h. The reaction mixture was directly loaded on an ODS column, and chromatographed with a stepwise elution of H_2O/MeOH (from 100/0 to 0/100). Fractions eluted with H_2O/MeOH (40/60) were combined and concentrated *in vacuo*. A portion of the residue (27.4 mg of 54.7 mg) was subjected to reversed-phase HPLC (PEGASIL ODS SP100, ϕ 20 × 250 mm, H_2O/MeOH (from 70/30 to 0/100)) to give **5** (15.0 mg, 50%): UV (MeOH) λ_{max} (log ε) 224 (5.02), 275 (4.63), 453 (4.31), 479 (4.30) nm; IR (neat) 3287, 2936, 2873, 1749, 1636, 1605, 1540, 1507, 1457, 1319, 1180, 1111, 846 cm^{-1}; ^1H NMR (CD_3OD, 500 MHz) δ 8.09 (d, J = 1.8 Hz, 1H), 7.78 (d, J = 7.7 Hz, 2H), 7.73 (br d, J = 8.0 Hz, 1H), 7.49 (dd, J = 7.3, 7.3 Hz, 1H), 7.42 (dd, J = 7.3, 7.3 Hz, 2H), 7.12 (d, J = 8.2 Hz, 1H), 6.67 (d, J = 8.8 Hz, 2H), 6.66 (d, J = 2.2 Hz, 2H), 6.53 (dd, J = 8.5, 2.5 Hz, 2H), 3.70 (br, 2H), 3.62-3.54 (ovl, 12H), 3.45 (t, J = 6.9 Hz, 2H), 1.89 (tt, J = 6.3, 6.3 Hz, 2H), 1.85 (tt, J = 6.5, 6.5 Hz, 2H); ^{13}C NMR (CD_3OD, 125 MHz) δ 182.5, 171.2, 170.2, 161.7, 154.3, 149.2, 142.2, 135.8, 132.5, 131.6, 130.4, 129.6, 129.3, 128.3, 125.9, 120.0, 113.8, 111.6, 103.5, 71.5-71.0, 70.3, 70.3, 43.7, 38.7, 30.6, 30.4; HRMS (ESI) *m/z* 736.2294 $[M + Na]^+$ calcd for $C_{38}H_{39}N_3NaO_9S$, 736.2299.

Synthesis of compound 6.

(E)-2-(6-hydroxy-3-oxo-3H-xanthen-9-yl)-5-(3-(1-oxo-1-phenyl-6,9,12-trioxa-2-azapentadecan-15-yl)-2-propylguanidino)benzoic acid (**6**)

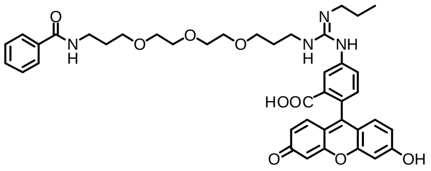

The thiourea compound **5** (7.6 mg, 0.0107 mmol), 0.2 mL of DMF/H_2O (1/1), and 0.8 mL of *n*-propylamine (9.73 mmol, 909 eq) were mixed and stirred in a brown microtube at room temperature for 3 weeks. The reaction mixture was directly loaded on an ODS column, and chromatographed with a stepwise elution of H_2O and MeOH. Fractions eluted with MeOH were combined and concentrated *in vacuo*. The residue (12.9 mg) was subjected to reverse-phase HPLC (PEGASIL ODS SP100, ϕ 20 × 250 mm, H_2O/MeOH (from 70/30 to 0/100)) to give **6** (2.5 mg, 31%): UV (MeOH) λ_{max} (log ε) 227 (4.84), 455(4.41), 482 (4.42) nm; IR (neat) 3211, 2928, 2871, 1759, 1636, 1608, 1548, 1504, 1455, 1307, 1180, 1112, 849 cm^{-1}; ^1H NMR (CD_3OD, 500 MHz) δ 7.82 (d, J = 2.1 Hz, 1H), 7.78 (d, J = 8.0 Hz, 2H), 7.48 (ovl, 1H),

7.48 (ovl, 1H), 7.42 (dd, *J* = 7.4, 7.4 Hz, 2H), 7.28 (d, *J* = 8.2 Hz, 1H), 7.07 (br d, *J* = 9.0 Hz, 2H), 6.68 (d, *J* = 1.9 Hz, 2H), 6.62 (dd, *J* = 2.2, 9.0 Hz, 2H), 3.60 (ovl), 3.63-3.56 (ovl), 3.55 (t, *J* = 6.4 Hz, 2H), 3.48 (br, 2H), 3.44 (t, *J* = 7.1 Hz, 2H), 3.31(ovl), 1.92 (br, 2H), 1.85 (tt, *J* = 6.5, 6.5 Hz, 2H), 1.70 (br q, *J* = 7.4 Hz, 2H), 1.01 (t, *J* = 7.5 Hz, 3H); ^{13}C NMR (CD_3OD, 125 MHz) δ 156.4, 155.8, 172.2, 170, 158.1, 138.7, 135.8, 132.8, 132.2, 131.2, 129.7, 128.4, 128.3, 125.4, 120.3, 114.9, 104.1, 71.5-71.1, 70.3, 45.2, 41.0, 38.9, 30.7, 29.7, 23.6, 11.7; HRMS (ESI) *m/z* 739.3334 $[M + H]^+$ calcd for $C_{41}H_{47}N_4O_9$, 739.3338.

Synthesis of probe 8.

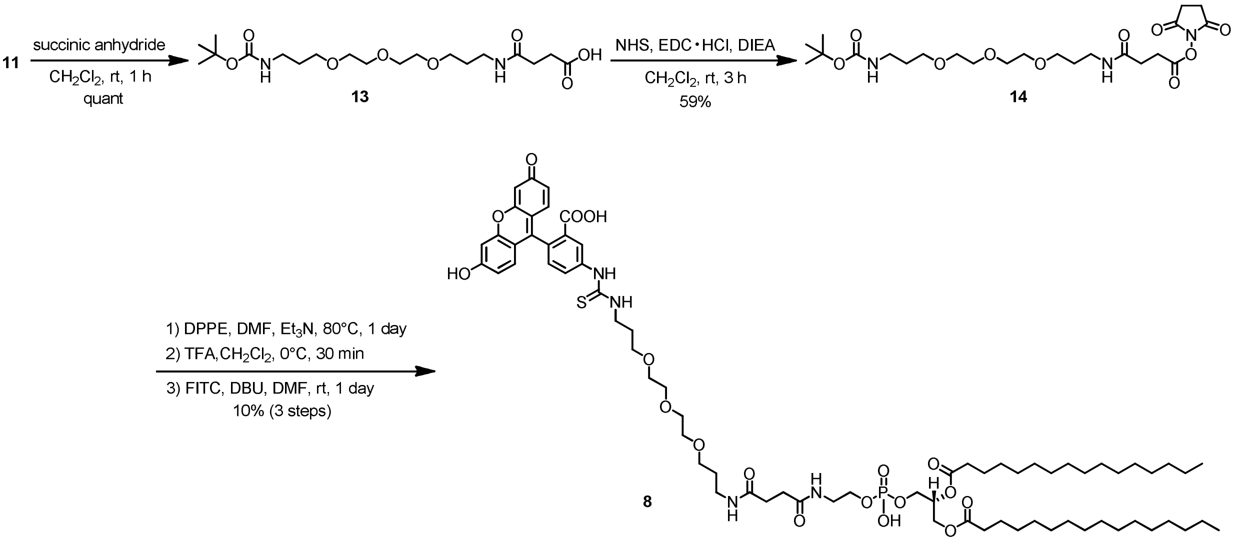

2,2-dimethyl-4,20-dioxo-3,9,12,15-tetraoxa-5,19-diazatricosan-23-oic acid (**13**)

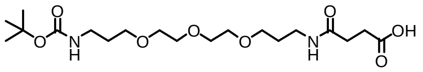

To a stirred solution of **11** (523.2 mg, 1.63 mmol) in CH_2Cl_2 (30 mL) was added succinic anhydride (338.0 mg, 3.38 mmol). After being stirred at room temperature for 1 h, the reaction was quenched with 5% aqueous citric acid. The mixture was extracted with $CHCl_3$, dried over Na_2SO_4, and concentrated *in vacuo* to afford a sodium salt of **13** as a yellowish blown oil (807.4 mg, quant): IR (neat) 3333, 2927, 2871, 1784, 1697, 1654, 1524, 1392, 1366, 1168, 1098, 749 cm^{-1}; ^{1}H NMR ($CDCl_3$, 500 MHz) δ 3.41 (br, 4H), 3.37 (br, 4H), 3.30-3.29 (ovl, 4H), 3.10 (dt, *J* = 6.3, 6.3 Hz, 2H), 2.96 (br, 2H), 2.41 (t, *J* = 6.9 Hz, 2H), 2.27 (t, *J* = 6.9 Hz, 2H), 1.57-1.50 (ovl, 4H), 1.20 (s, 9H); ^{13}C NMR ($CDCl_3$, 125 MHz) δ 175.1, 172.4, 156.2, 78.8, 70.3, 70.3, 70.0, 69.9, 39.4, 69.2, 38.2, 37.6, 30.7, 29.7, 29.5, 28.8, 28.4; HRMS (ESI) *m/z* 421.2548 $[M + H]^+$ calcd for $C_{19}H_{37}N_2O_8$, 421.2544.

2,5-dioxopyrrolidin-1-yl 2,2-dimethyl-4,20-dioxo-3,9,12,15-tetraoxa-5,19-diazatricosan-23-oate (**14**)

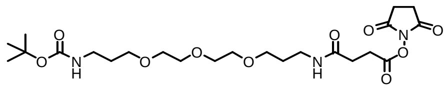

To a stirred solution of **13** (339.4 mg, 0.912 mmol) in CH_2Cl_2 (20 mL) were added EDC·HCl (261.6 mg, 1.36 mmol) and DIEA (238.6 μL, 1.37 mmol). After being stirred at room temperature for 30 min, NHS (158.6 mg, 1.38 mmol) was added. After being stirred for 3 h, the reaction was quenched with 5% aqueous citric acid. The mixture was extracted with $CHCl_3$. The organic layer was washed with saturated aqueous $NaHCO_3$ and brine, dried over Na_2SO_4, and concentrated *in vacuo* to afford **14** as a light brown oil (233.8 mg, 59%): IR (neat) 3327, 2929, 2870, 1814, 1784, 1737, 1697, 1660, 1529, 1365, 1170, 1070, 752, 647 cm^{-1}; ^1H NMR ($CDCl_3$, 500 MHz) δ 3.45 (br, 4H), 3.40 (br, 4H), 3.38-3.33 (ovl, 4H), 3.15 (dt, J = 5.7, 5.7 Hz, 2H), 3.00 (br, 2H), 2.77 (t, J = 6.9 Hz, 2H), 2.65 (s, 4H), 2.39 (t, J = 6.9 Hz, 2H), 1.61-1.53 (ovl, 4H), 1.24 (s, 9H); ^{13}C NMR ($CDCl_3$, 125 MHz) δ 177.5, 169.9, 169.3, 168.3, 156.0, 78.7, 70.4, 70.1, 70.0, 69.6, 69.3, 38.3, 37.8, 30.3, 29.6, 28.9, 28.4, 26.7, 25.5; HRMS (ESI) *m/z* 540.2521 $[M + Na]^+$ calcd for $C_{23}H_{39}N_3NaO_{10}$, 540.2528.

5-(3-(1-((((R)-2,3-bis(palmitoyloxy)propoxy)(hydroxy)phosphoryl)oxy)-4,7-dioxo-12,15,18-trioxa-3,8-diazahenicosan-21-yl)thioureido)-2-(6-hydroxy-3-oxo-3H-xanthen-9-yl)benzoic acid (**8**)

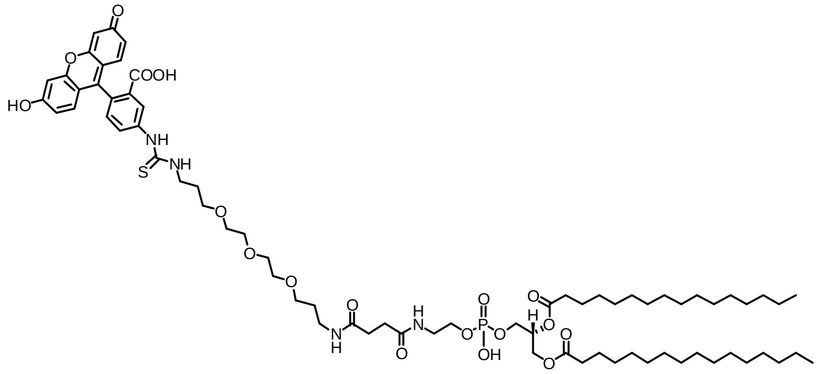

To a stirred solution of **14** (29.9 mg, 0.0578 mmol) in DMF (2 mL) were added 1,2-dipalmitoyl-*sn*-glycero-3-phosphoethanolamine (DPPE) (40.48 mg, 0.0585 mmol) and Et_3N (8.06 μL, 0.0578 mmol). After being stirred at 80 ºC for 1 day, the reaction mixture was concentrated *in vacuo*. The residue was dissolved in CH_2Cl_2, and then TFA (2 mL) was added dropwise at 0 ºC. After the reaction mixture was stirred at 0 ºC for 30 min, the mixture was concentrated *in vacuo* with toluene to give a residue containing **15**. This crude **15** (145.05 mg, 0.131 mmol), FITC (51.48 mg, 0.132 mmol), 0.5 mL of DMF, and DBU (92.8 μL, 0.620 mmol) were mixed and stirred in a brown microtube at room temperature for 1 day. The mixture was directly loaded on an ODS column and chromatographed with a stepwise elution of H_2O/MeOH (from 100/0 to 0/100). Fractions eluted with H_2O/MeOH (10/90 to 0/100) were combined and concentrated *in vacuo*. The residue was subjected to reverse-phase HPLC (COSMOSIL $5C_8$-MS, ϕ 20 × 250 mm, H_2O/MeOH (from 20/80 to 0/100)) to give **8** (8.38 mg, 10%, 4 steps): $[\alpha]_D^{20}$ = -23.7 (c 0.76, CH_3OH); UV (MeOH) λ_{max} (log ε) 220 (4.61), 275 (4.17), 453 (3.83), 479 (3.84) nm; IR (neat) 3264, 3069, 2917, 2850, 1740, 1643, 1581, 1467, 1325, 1208, 1106 cm^{-1}; ^1H NMR (CD_3OD, 500 MHz) δ 8.04 (s, 0.13H), 7.97 (s, 0.87H), 7.90 (d, J = 8.3 Hz, 0.13H), 7.74 (d, J = 7.9 Hz, 0.87H), 7.18 (m, 1H), 7.08 (m, 2H), 6.61-6.58 (ovl, 4H), 5.21 (m, 1H), 4.42 (dd, J = 12.0, 3.3 Hz, 1H), 4.17 (dd, J = 12.0, 6.8 Hz, 1H),

3.97 (dd, *J* = 5.6, 5.6 Hz, 2H), 3.89 (dt, *J* = 5.9, 5.9 Hz, 2H), 3.74 (br, 2H), 3.63-3.55 (ovl, 10H), 3.52 (t, *J* = 6.0 Hz, 2H), 3.39 (t, *J* = 5.6 Hz, 2H), 3.31 (ovl, 2H), 2.47 (m, 4H), 2.31 (m, 4H), 2.01 (tt, *J* = 6.0, 6.0 Hz, 2H), 1.93 (tt, *J* = 6.2, 6.2 Hz, 2H), 1.58 (m, 4H), 1.28 (ovl, 48H), 0.89 (ovl, 6H); HRMS (ESI) *m/z* 1383.7409 $[M + H]^+$ calcd for $C_{72}H_{112}N_4O_{18}PS$, 1383.7424.

Supplementary figures

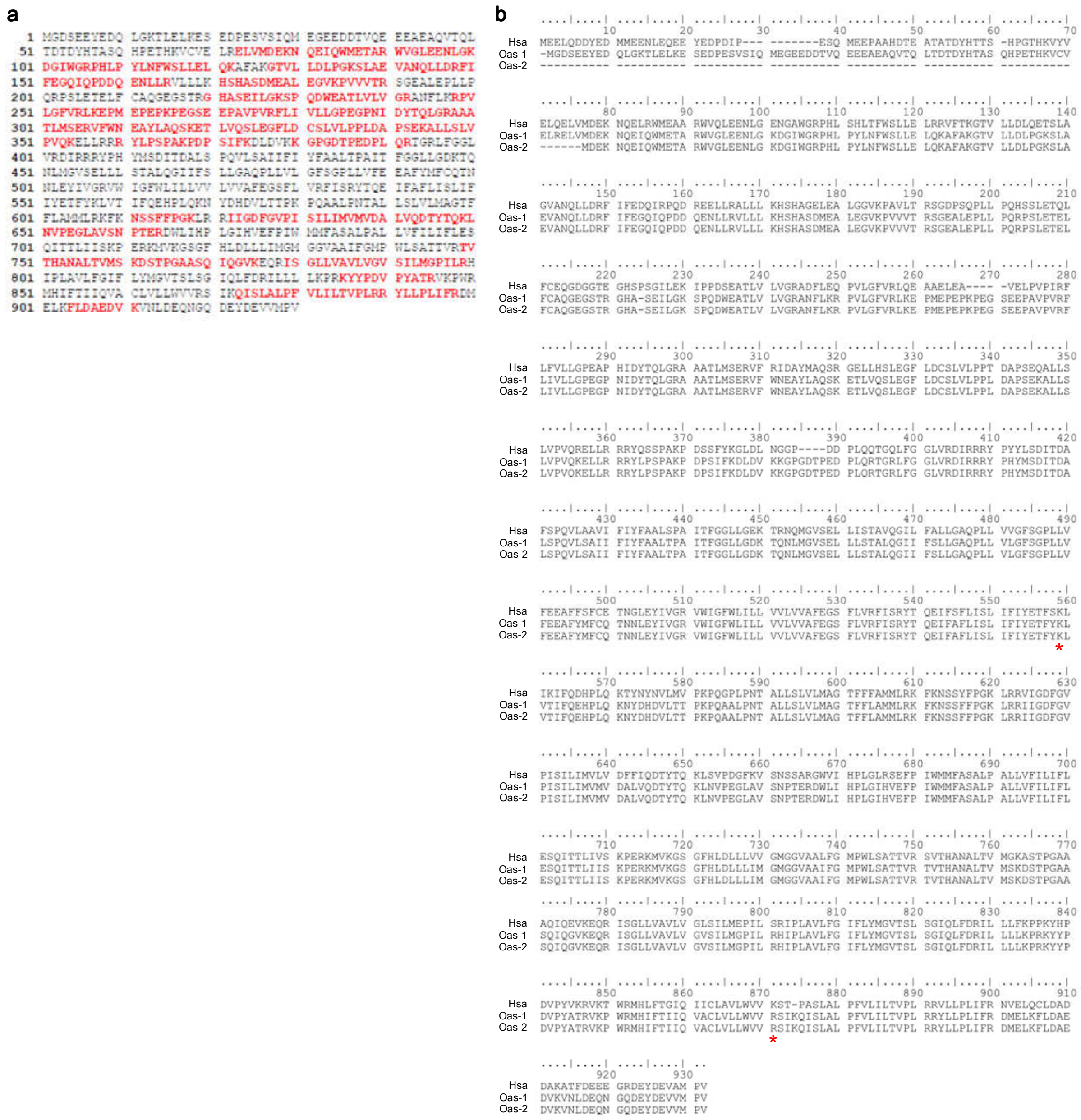

Figure S1. The amino acid sequence of the anion transporter band 3. (a) The 100kDa protein band was digested in gel using trypsin, and analyzed by LC-MS/MS. The red sequences were identified by the MASCOT search engine. The sequence coverage was 44%. (b) The amino acid sequence of band 3 from *Homo sapiens* (Hsa) and *Ovis aries* (Oas) are compared. Two variants for *O. aries* are shown. Asterisks show DIDS sensitive lysines, Lys539 and Lys 851 (Hsa), and Lys 557 (Oas). Lys851 (Hsa) is not conserved in *O. aries* (Arg869). In the SDS-PAGE analysis, we detected two bands, which might be valiants of band 3 consisting of 930 and 855 amino acids. In fact, the tagging of both bands was inhibited by DIDS.

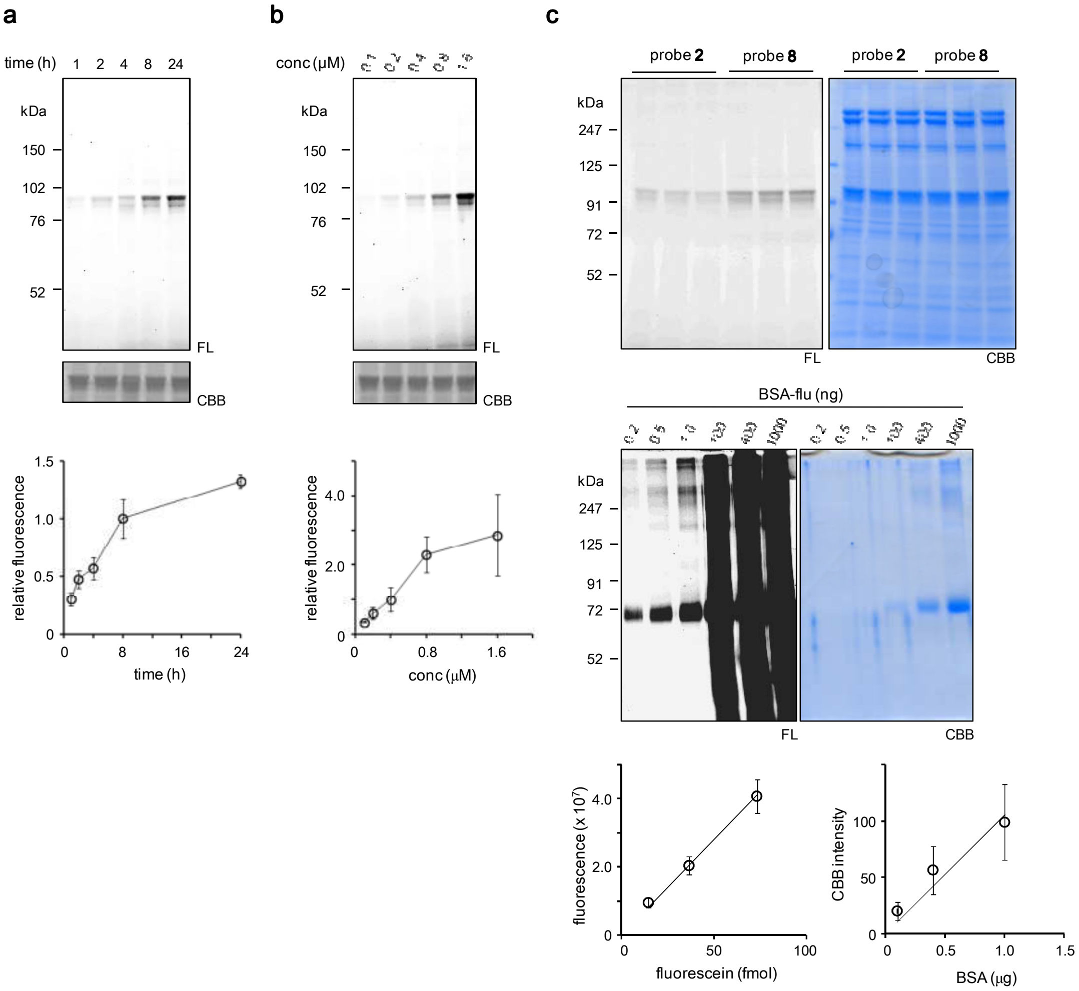

Figure S2. Kinetics of tagging of band 3 by the cholesterol probe 2. (a) Time course analysis of the tagging. Erythrocytes were treated with the cholesterol probe **2** (400 nM) at 37 ºC for indicated time. Cells were collected, lysed, and analyzed by SDS-PAGE. The fluorescence signal of fluorescein and the CBB image of band 3 are shown. (b) Concentration dependence of the tagging. A variety of concentrations of probe **2** was incubated with erythrocytes for 8 h. Gel images were obtained as in (a). Fluorescence intensities relative to that obtained using 400 nM of the probe by 8 h incubation are shown. (c) Quantitative analysis of the tagging by probe **2** and probe **8**. The amount of the tagged protein and the amount of all band 3 protein were calculated using fluorescein-labeled BSA (Invitrogen). When erythrocytes (10e8 cells/mL) were treated with probe **2** (400 nM) for 8 h, the amount of the tagged protein and all band 3 protein were 6.68 fmol and 9.12 pmol (0.95 μg), respectively, corresponding to the tagging yield of 0.073%. Probe **8** (400 nM, 8 h) tagged 18.9 fmol of band 3 protein (9.13 pmol, 0.95 μg), corresponding to the tagging yield of 0.21%. (a-c) Means ± SD of three experiments are shown.

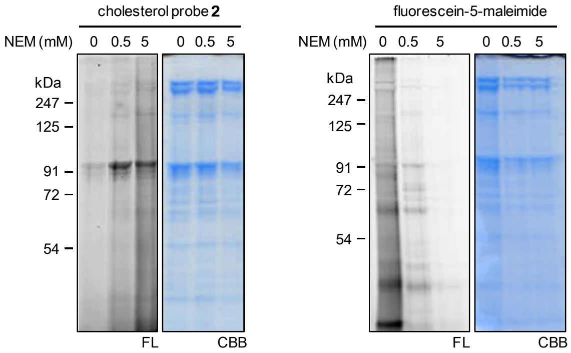

Figure S3. Effect of *N*-ethylmaleimide against tagging of band 3 by the cholesterol probe 2. Erythrocytes (10e8 cells/mL) were treated with *N*-ethylmaleimide (0.5 or 5 mM in 0.5% DMF) on ice and incubated at 37 ºC for 1 h with continuous mixing. After incubation, erythrocytes were washed with phosphate buffer (1 mL) twice, suspended in 1 mL of phosphate buffer, and treated with probe **2** or fluorescein-5-maleimide at 37 ºC for 8 h.

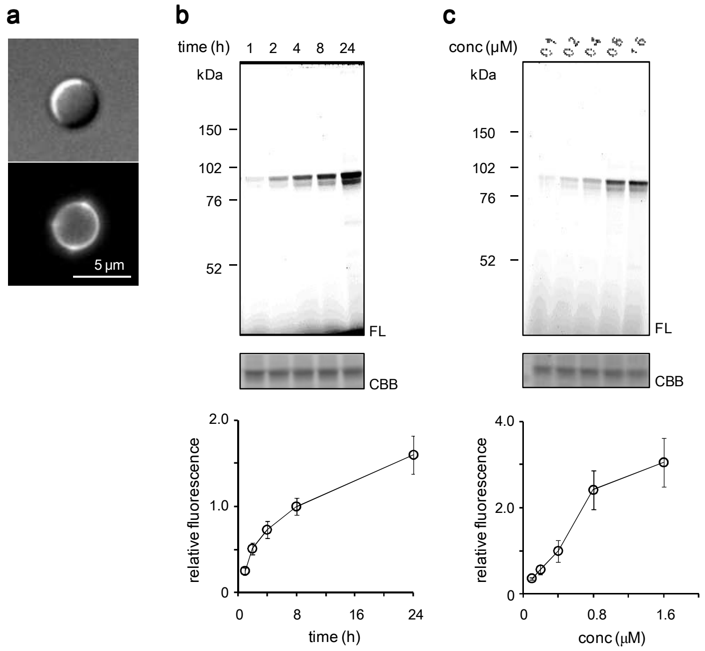

Figure S4. Kinetics of tagging of band 3 by the DPPE probe 8. (a) Binding of probe **8** to the erythrocyte. Bright field (DIC) and fluorescent images are shown. (b) Time course analysis of the tagging. Erythrocytes were treated with the DPPE probe **8** (400 nM) at 37 ºC for indicated times. (c) Concentration dependence of the tagging. A variety of concentrations of probe **8** was incubated with erythrocytes for 8 h. Gel images were obtained as in **Figure S2**. Fluorescence intensities relative to that obtained using 400 nM of the probe by 8 h incubation are shown. (b-c) Means ± SD of three experiments are shown.

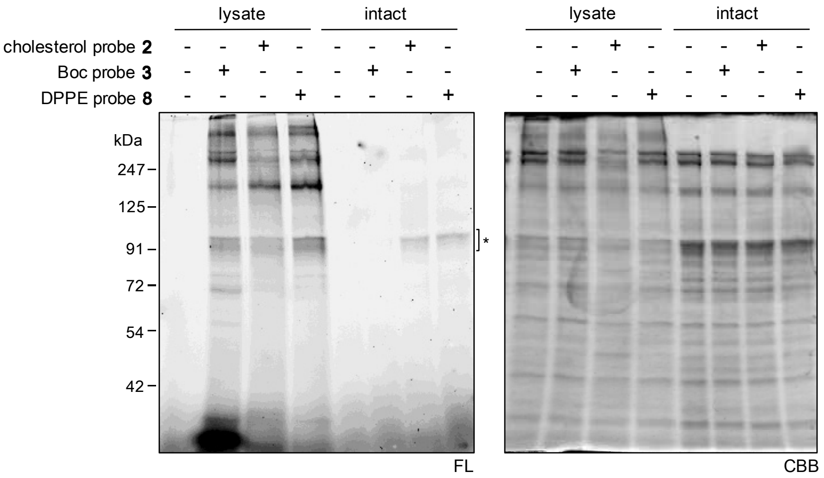

Figure S5. Chemical tagging of proteins in intact and lysed erythrocytes. Erythrocytes (10e8 cells in 200 μL PBS) were incubated with probes **2**, **3**, or **8** (1 μM) at 37 ºC for 8 h, in the presence or absence of triton X100 (1%). After incubation, erythrocytes were washed and suspended in 80 μL of SDS-PAGE sample buffer, and 15 μL of the sample was applied to SDS-PAGE. Lysed erythrocyte solution (80 μL) was mixed with 20 μL of SDS-PAGE sample buffer, and 30 μL of the sample was applied to SDS-PAGE. When cells were lysed by triton X-100, several proteins were tagged by not only lipid probes **2** and **8**, but also by the Boc probe **3**. In contrast, band 3 was tagged only by lipid probes **2** and **8** in the intact cells.

^{1}H NMR spectrum of **2** in CD_3OD (500 MHz).

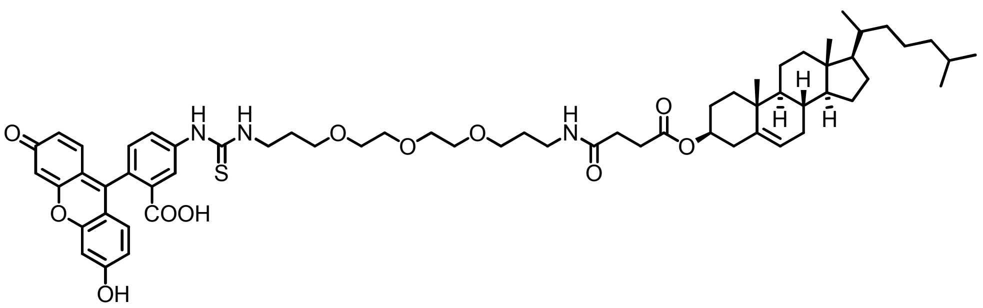

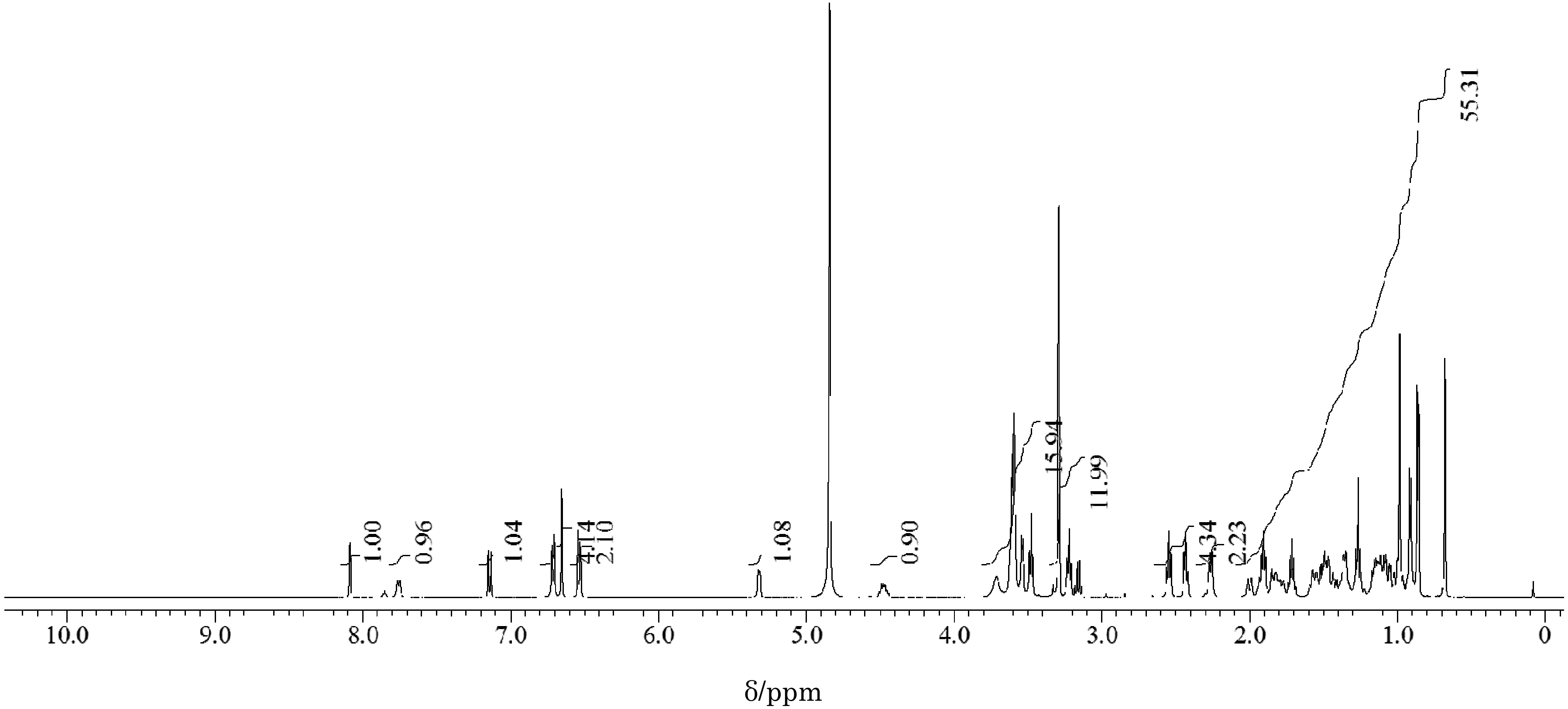

^{1}H NMR spectrum of **3** in CD_3OD (500 MHz).

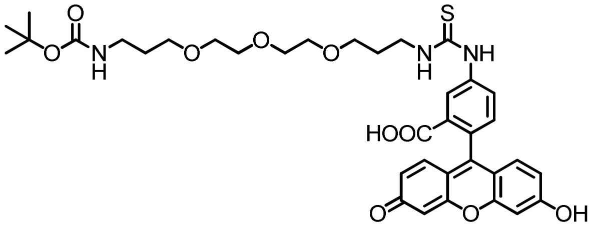

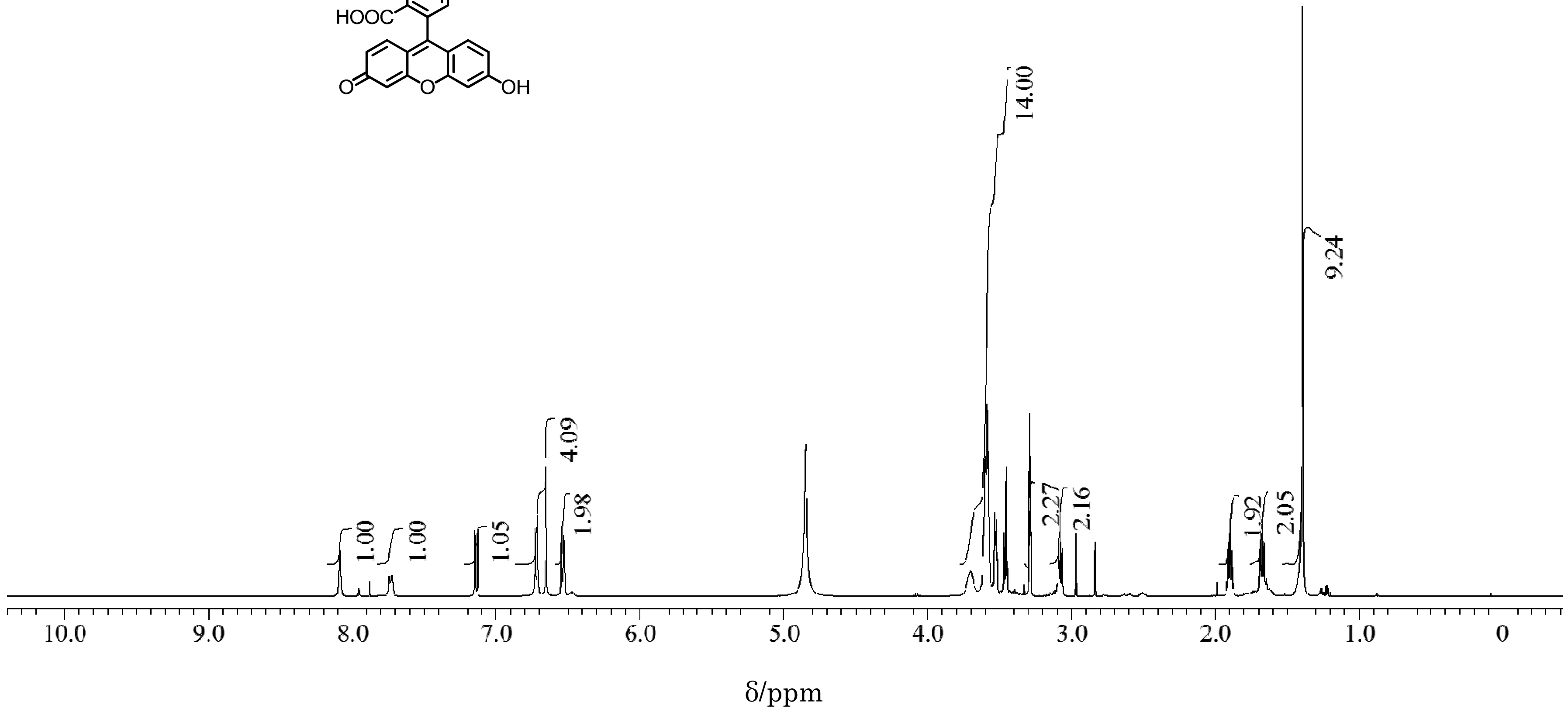

^{1}H NMR spectrum of **4** in CD_3OD (500 MHz).

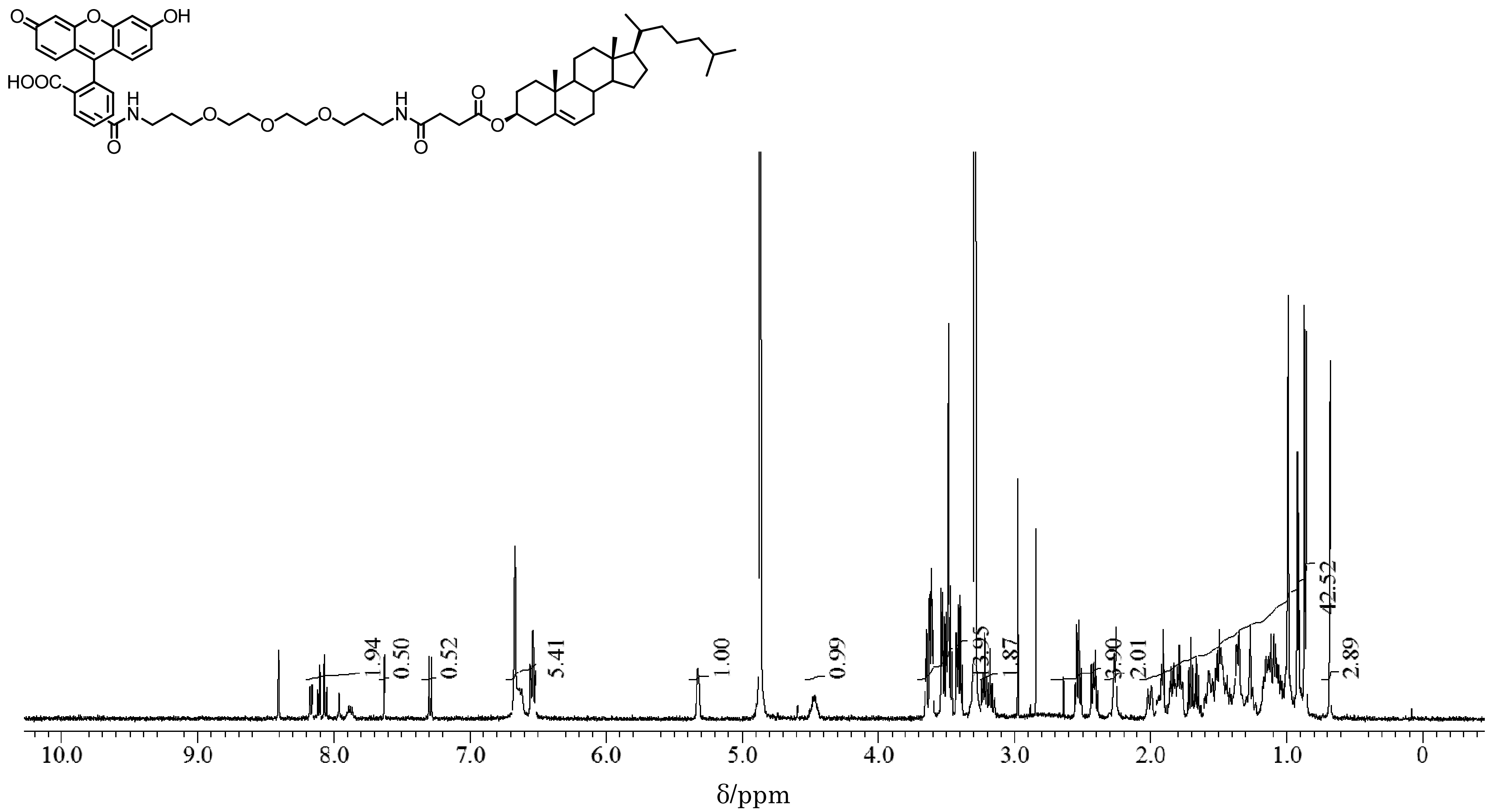

^1H NMR spectrum of **5** in CD_3OD (500 MHz).

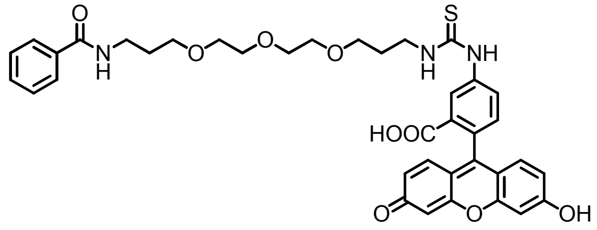

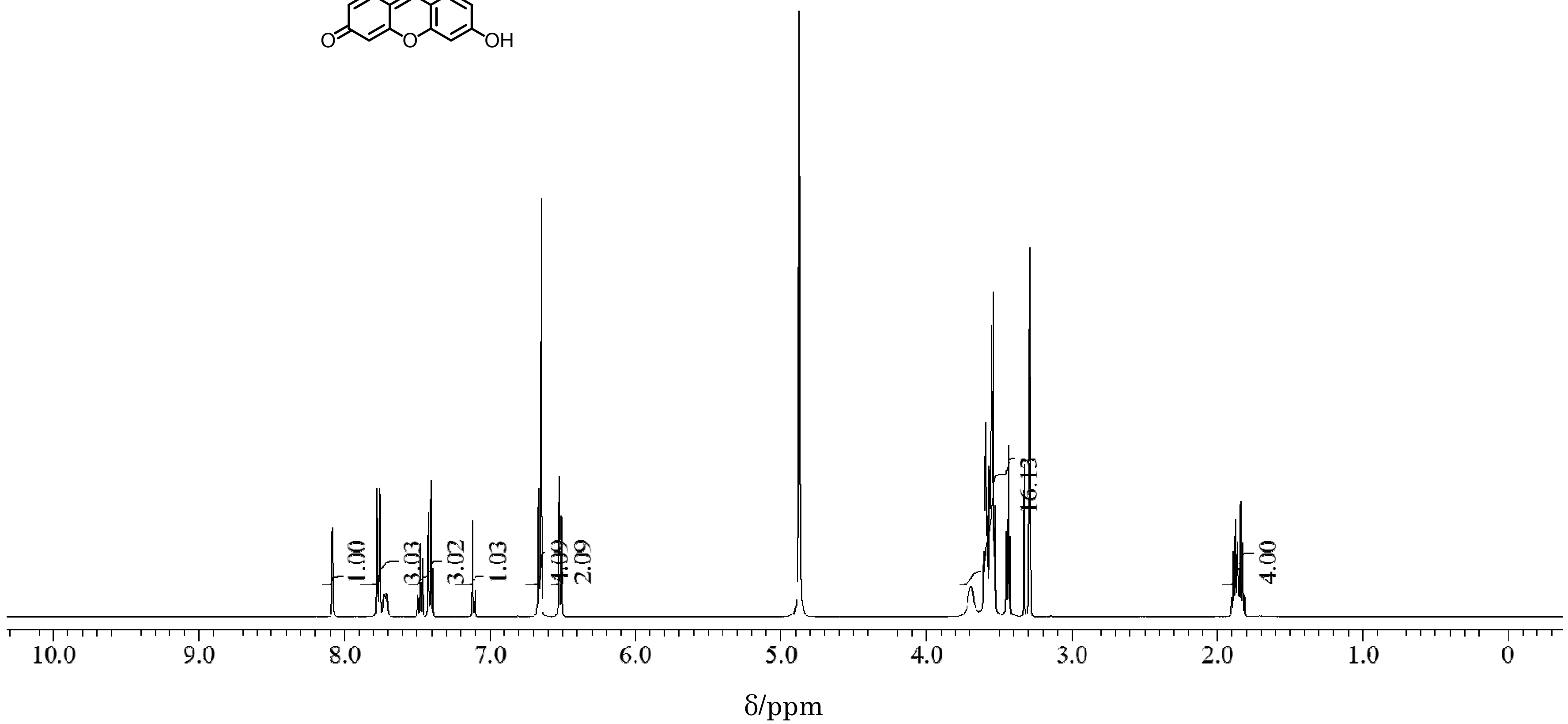

^{1}H NMR spectrum of **6** in CD_3OD (500 MHz).

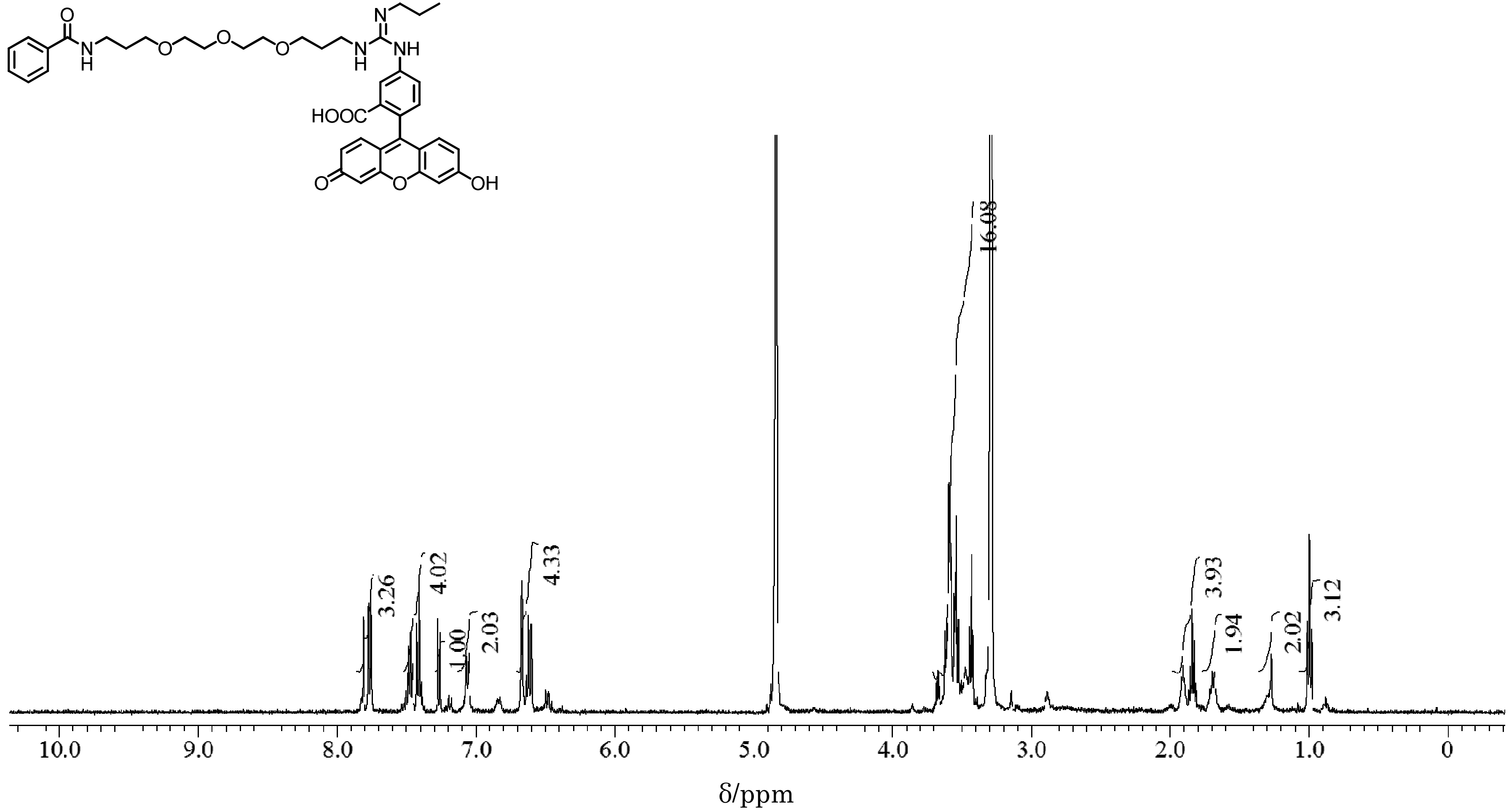

^1H NMR spectrum of **8** in CD_3OD (500 MHz).

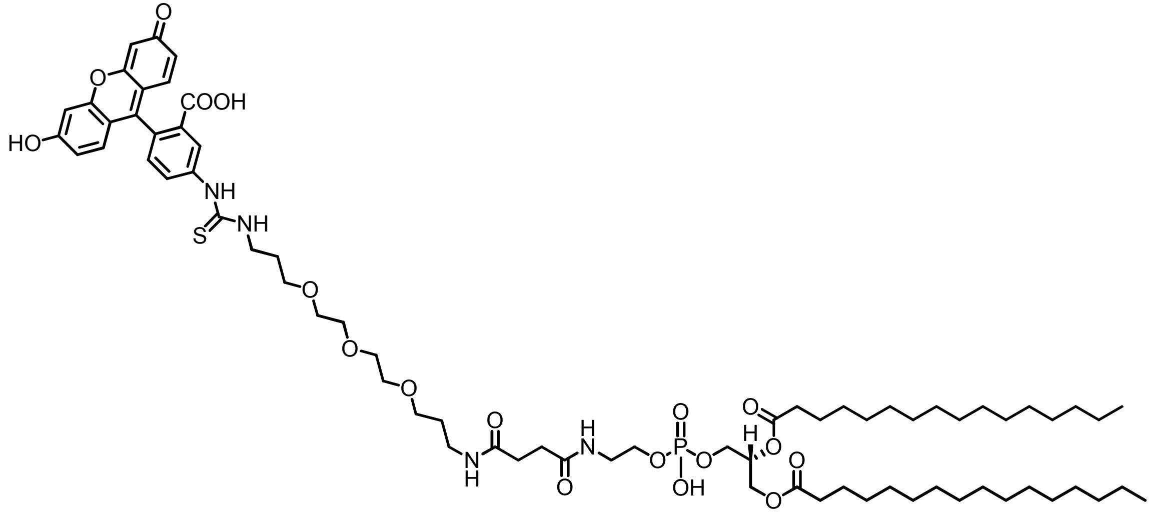

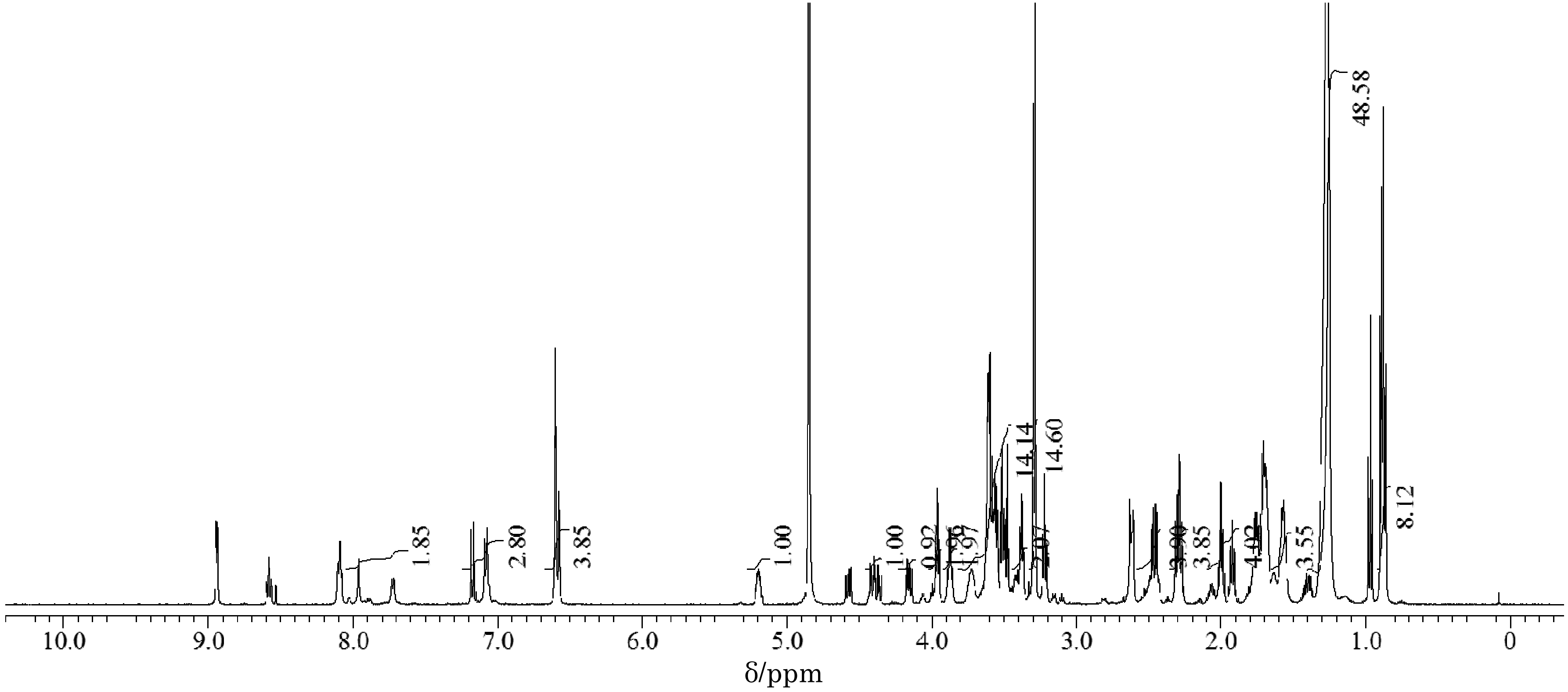

^1H NMR spectrum of **10** in $CDCl_3$ (500 MHz).

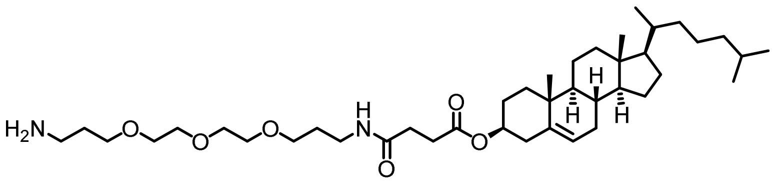

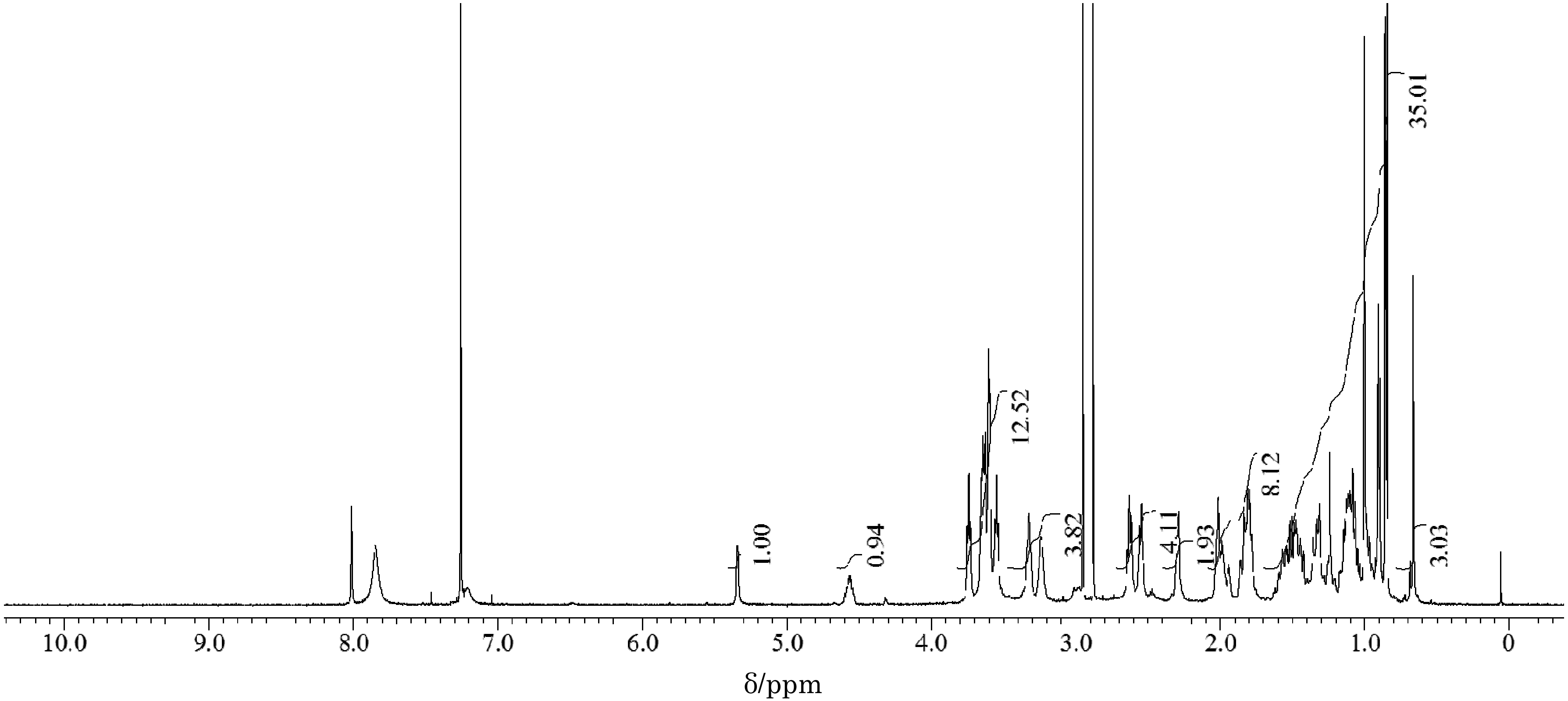

^{1}H NMR spectrum of **11** in $CDCl_3$ (500 MHz).

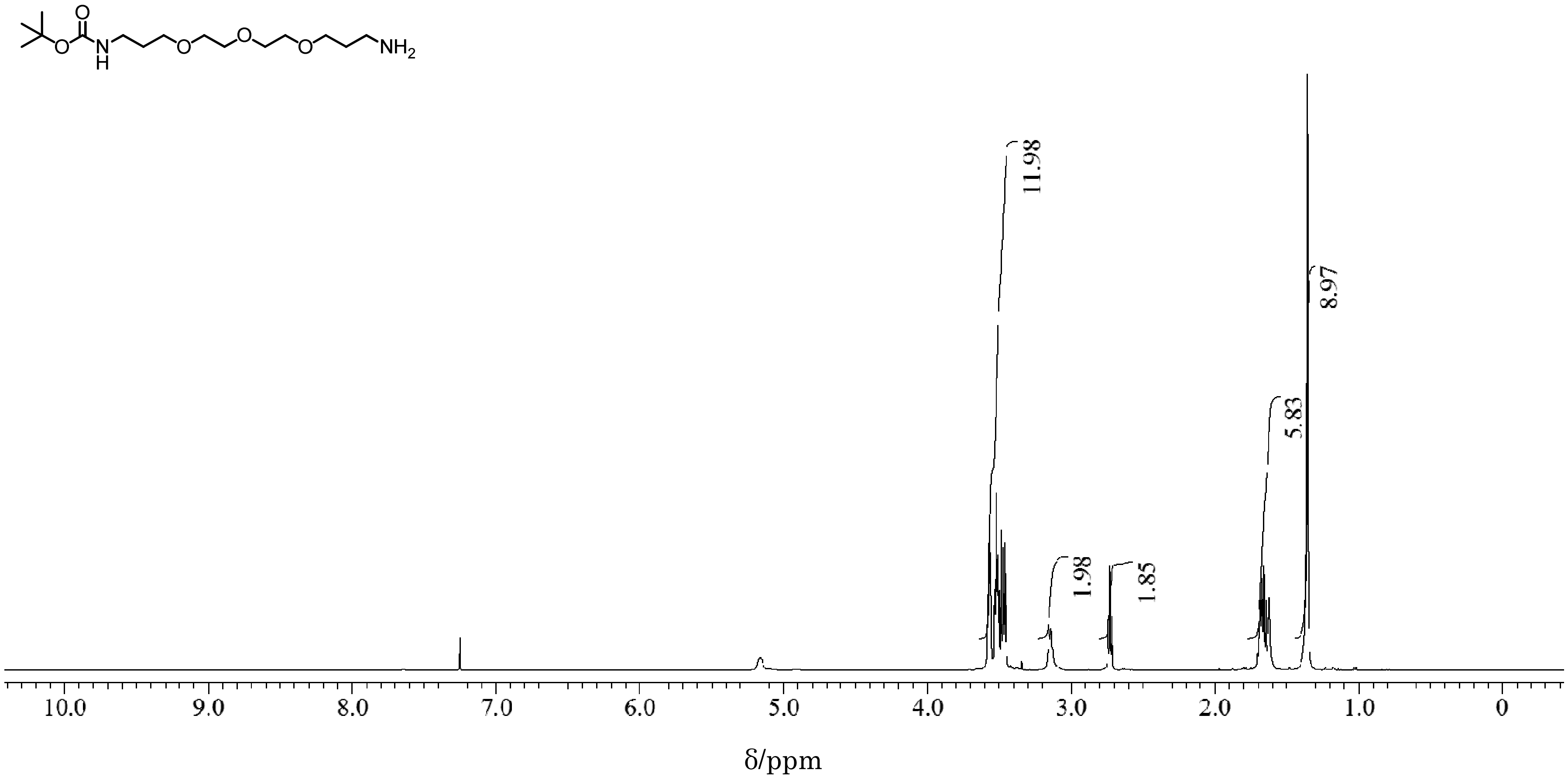

^{1}H NMR spectrum of **12** in CD_3OD (500 MHz).

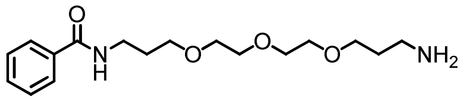

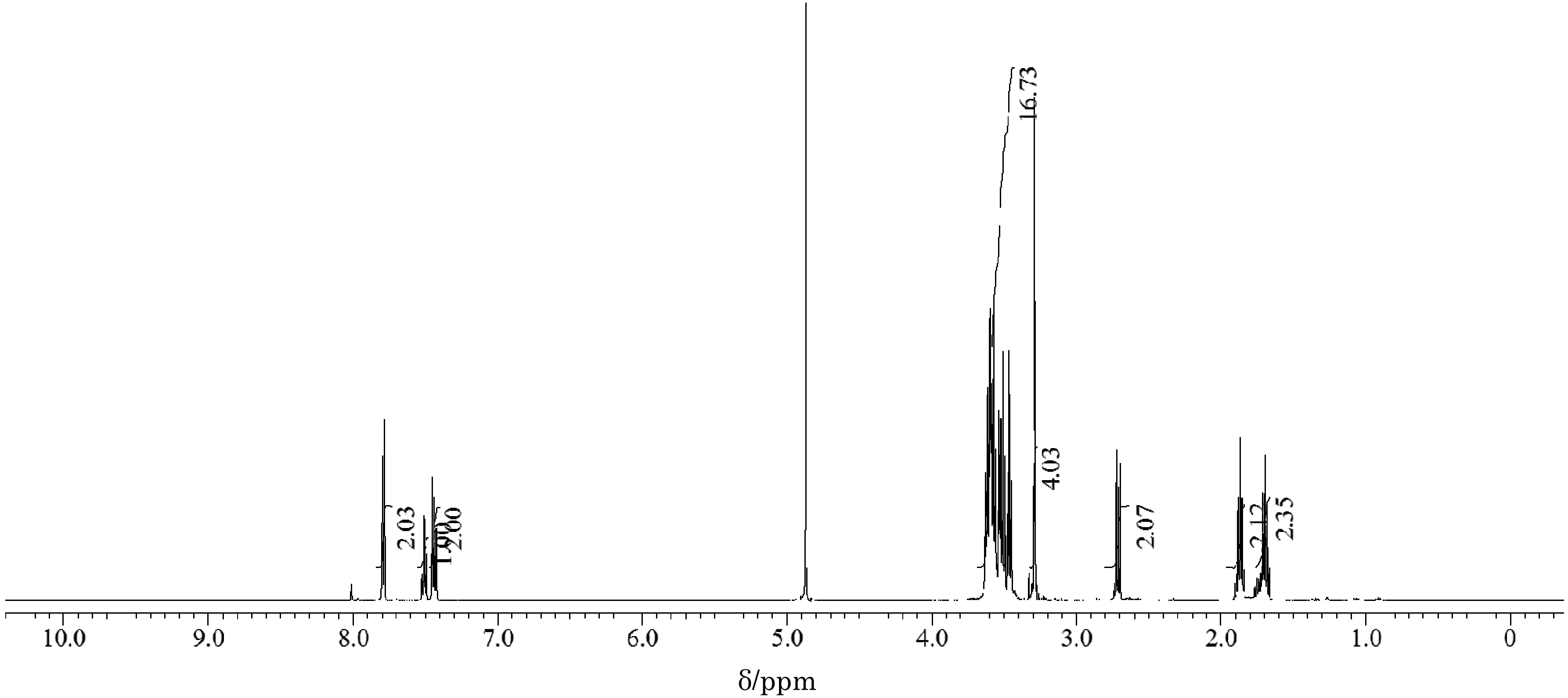

^{1}H NMR spectrum of **13** in $CDCl_3$ (500 MHz).

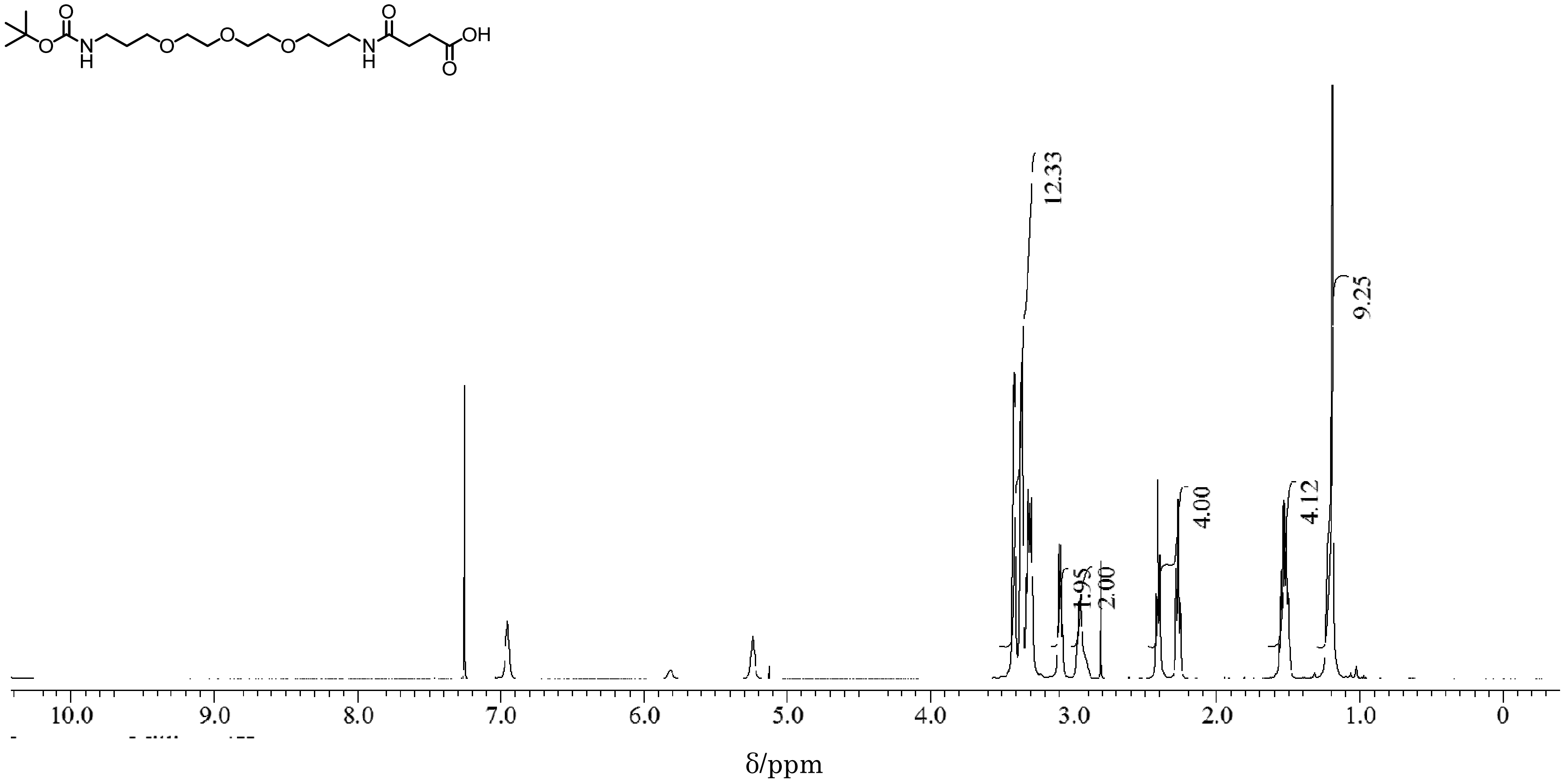

^{1}H NMR spectrum of **14** in $CDCl_3$ (500 MHz).

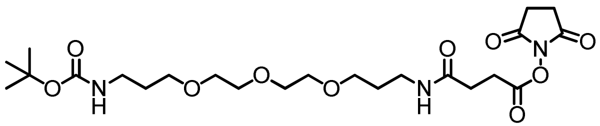

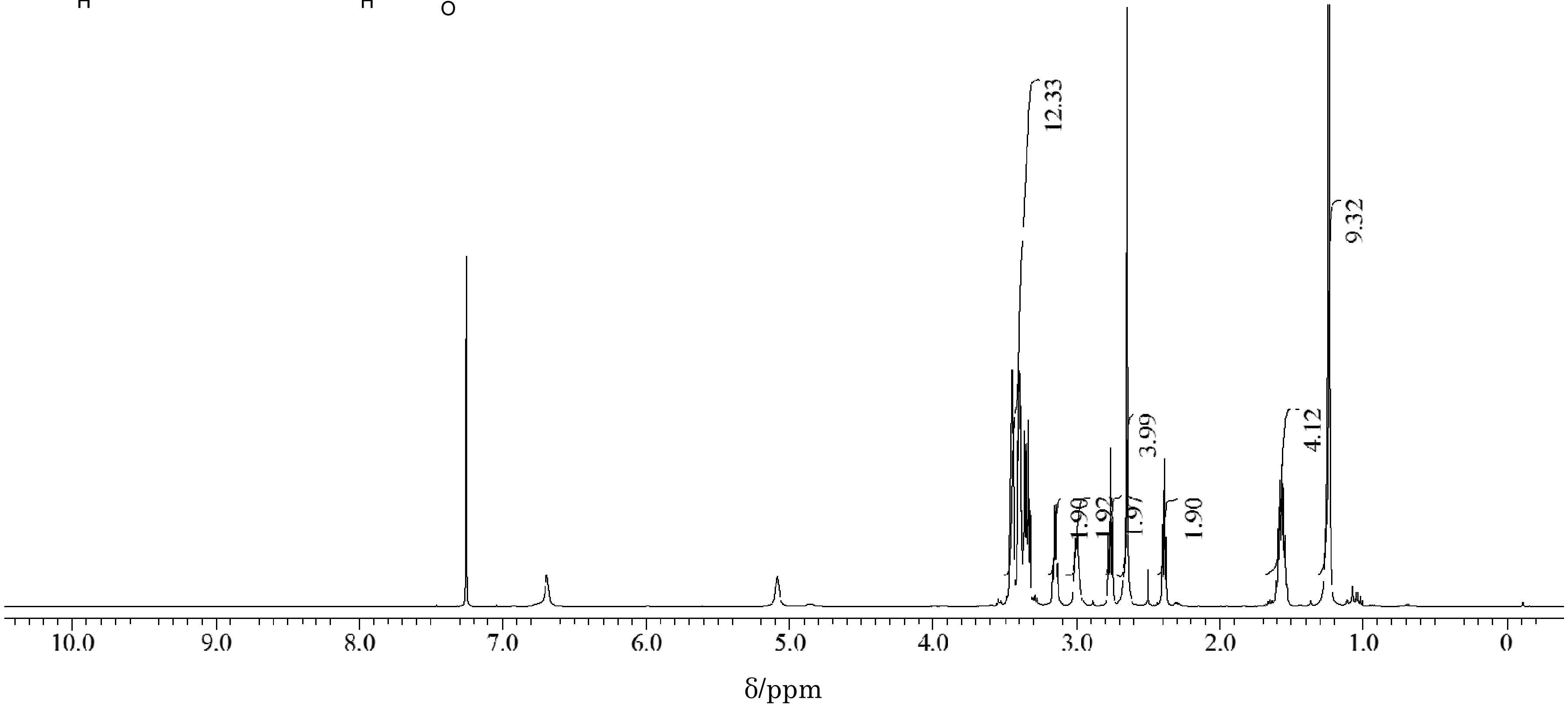

Supplement: Supplementary Information [file srep17427-s1.pdf]
